# Large-scale and innervated functional human gut tissues for transplantation via transient spheroid confinement

**DOI:** 10.1038/s41551-026-01688-6

**Published:** 2026-05-22

**Authors:** Holly M. Poling, Théo Noël, Akaljot Singh, Garrett W. Fisher, Konrad Thorner, Praneet Chaturvedi, Kalpana Nattamai, Kalpana Srivastava, Matthew R. Batie, Taylor Hausfeld, Amy L. Pitstick, Nicole E. Brown, Séverine Ménoret, Ignacio Anegon, Riccardo Barrile, Christopher N. Mayhew, Takanori Takebe, James M. Wells, Michael A. Helmrath, Maxime M. Mahe

**Affiliations:** 1 Division of Pediatric General and Thoracic Surgery, Cincinnati Children’s Hospital Medical Center, Cincinnati, OH USA; 2 Department of Biomedical Engineering, University of Cincinnati, Cincinnati, OH USA; 3 Center for Stem Cell and Organoid Medicine, Cincinnati Children’s Hospital Medical Center, Cincinnati, OH USA; 4 Nantes Université, Inserm, TENS UMR1235, The Enteric Nervous System in Gut and Brain Diseases, IMAD, Nantes, France; 5 Division of Developmental Biology, Cincinnati Children’s Hospital Medical Center, Cincinnati, OH USA; 6 Digital Experience Center, Cincinnati Children’s Hospital Medical Center, Cincinnati, OH USA; 7 Nantes Université, CHU Nantes, Inserm, CNRS, SFR Santé, Inserm UMS 016 CNRS UMS 3556, Nantes, France; 8 INSERM, Centre de Recherche en Transplantation et Immunologie UMR1064, Nantes Université, Nantes, France; 9 Division of Gastroenterology, Hepatology and Nutrition, Cincinnati Children’s Hospital Medical Center, Cincinnati, OH USA

**Keywords:** Stem-cell differentiation, Gastrointestinal system, Tissue engineering

## Abstract

Key limitations of current human gastrointestinal organoids include incomplete physiological maturation and the need for complex, time-consuming assembloid approaches to integrate a functional nervous system for transplantation. Here we present a confined culture system (CCS) method that generates large-scale, elongated and functional human small intestinal, colonic and gastric tissues with a de novo enteric nervous system (ENS). We use a 3D-printed scaffolding tray to restrict spheroid fusion and growth, promoting the spontaneous co-development of a functional ENS. Transcriptomic and electrophysiological data demonstrate selective neuromuscular function and the presence of excitatory and inhibitory neurons within the tissues. When compared to traditional methods, the CCS expedites tissue maturation for transplantation, yielding organoids that are up to ten times larger, reaching widths of 8 cm after 10 weeks, and exhibit enhanced engraftment rate. CCS organoids integrate and adapt to murine luminal environment while maintaining barrier integrity and functional capacity. The CCS methodology simplifies current protocols while accelerating the production of complex, functional and clinically relevant gut tissues.

## Abstract (web summary)

A confined culture system streamlines and accelerates the engineering of large-scale human gastrointestinal tissues that spontaneously develop a functional enteric nervous system, simplifying the production of complex, clinically relevant organs for transplantation.

## Main

The ability to culture and manipulate human pluripotent stem cells (hPSC) has advanced the generation of specific tissues^1^. Translational embryology has enabled ‘organogenesis in a dish’ in three-dimensional (3D) environments, fostering more physiologically relevant models such as organoids^2,3^. Defined by their self-assembly and renewal, organoids mimic native tissue, include correct cell types and replicate some organ functions^4^. These systems have been developed for many organs, including the gastrointestinal tract^5–10^, offering real-time visualization of organogenesis and bridging the gap between animal models and humans. hPSC-derived organoids hold immense potential for studying development, disease processes, cell-based therapies and personalized drug discovery.

Despite these advantages, current organoid technologies face limitations in mimicking physiological environments in vitro, often requiring in vivo transplantation for continued tissue differentiation. Some hPSC-derived gastrointestinal tissues exhibit incomplete maturation, limiting their transplantation^11^. We hypothesized that methods to promote cellular complexities would be required to support both in vitro and in vivo maturation that will approximate native developing tissues^11,12^. hPSC-derived cells undergo normal maturation, thus initially have embryonic or fetal-like characteristics^12,13^. Methods to further develop these tissues are required to achieve the much-desired mature tissues to model human bowel. Using existing protocols, human intestinal organoids (HIOs) grow individually, reaching ~1 mm after 28 days in culture, expanding to ~1 cm with transplantation^14–16^. However, their spherical shape differs from the intestinal tube. We introduced strain into transplanted HIOs to induce maturation and enterogenesis, producing elongated tissues resembling the postnatal gut^12^. Recent methods to generate hindgut (human colonic organoids, HCO) and foregut (human gastric organoids, HGO; human antral gastric organoids, HaGO) organoids have furthered understanding of gastrointestinal development and disease, although traditional culturing results in low engraftment rates and small sizes compared to HIOs^7,11^. Initial attempts to correct this were achieved in an assembloid approach by adding both splanchnic mesenchyme and neural crest cells^11^.

In this study, we focused on simplifying the methods required to achieve robust engraftment using a confined culture system (CCS). We designed a specialized scaffolding tray to provide a permissive environment for spheroid fusion (the 3D rounded structures that form from 2D monolayers upon differentiation, which are typically collected and re-plated for maturation into organoids). Loading a critical mass of spheroids into the scaffolding trays yielded longitudinal organoid structures. This device combined with 3D culture techniques to prime organoids successfully enhanced tissue growth and maturation, yielding organoids ten times larger than those in previous protocols. Notably, a de novo enteric nervous system (ENS) emerged, displaying selective neuronal excitation and contractile functions. Similar results were observed in colonic and gastric organoids. These structures readily engrafted, exhibited enhanced maturation and developed a functional ENS. Here we present a scalable organoid generation method emphasizing cellular confinement, advancing the production of complex organs for clinical applications.

## Results

### Implementation of a scaffolding tray for organoid generation

**Fig. 1:**
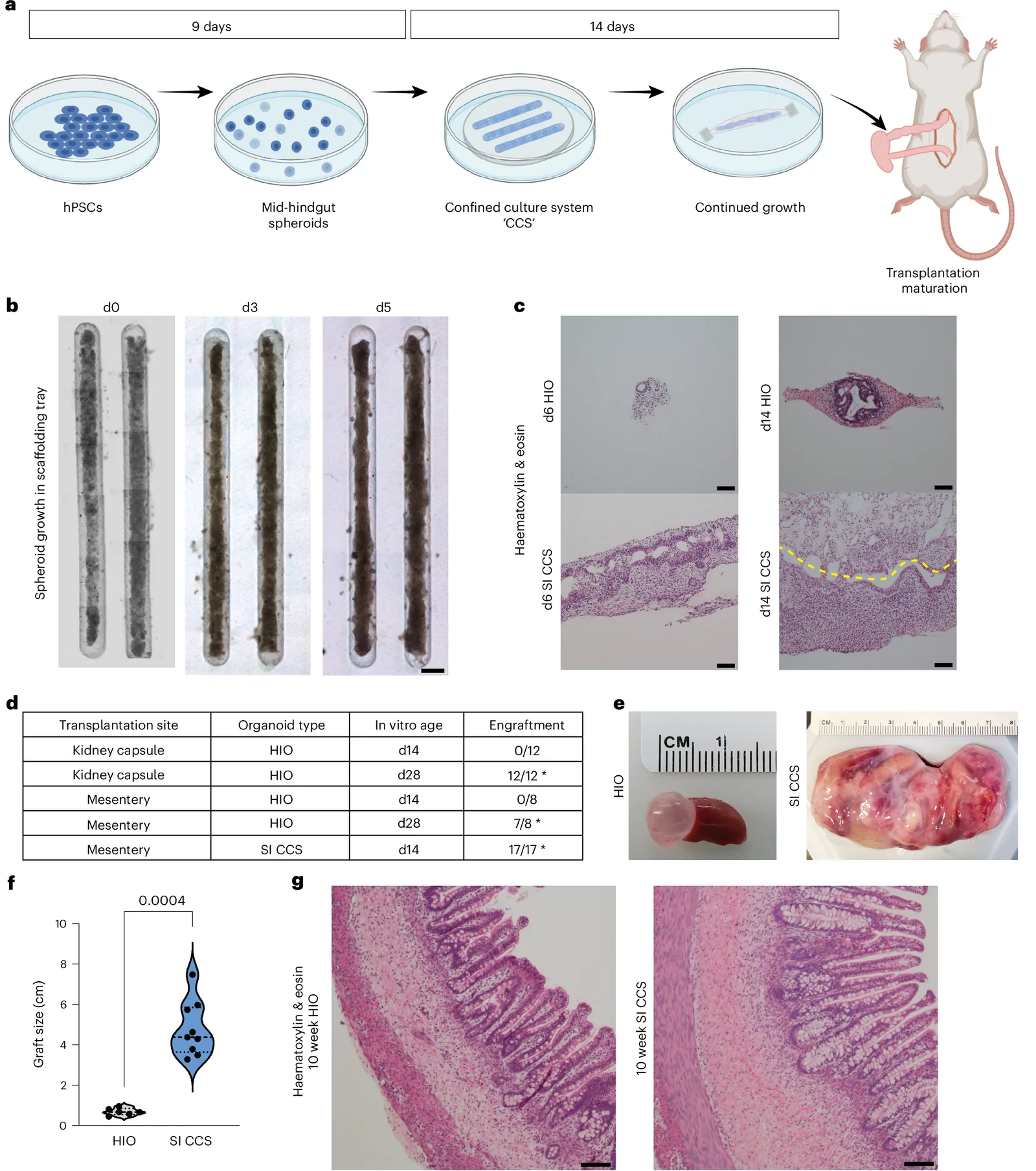
Employing a confinement strategy for the in vitro growth of small intestinal organoids. **a**, Experimental schematic. hPSCs were cultured and differentiated into mid-hindgut spheroids following published protocols, collected and seeded within the confinement lanes of a specialized scaffolding tray, allowed to grow for 6 days, then removed from the tray and re-plated. Culture was continued until d14 at which time the elongated organoids were transplanted. **b**, Tile scan images of spheroids growing within the scaffolding tray confinement lanes at d0, d3 and d5 of culture. Scale bar, 1 mm. **c**, Representative images of haematoxylin and eosin (H&E)-stained sections of conventionally generated HIOs and SI CCS at d6 and d14 of culture. The yellow dashed line delineates the intestinal lumen. Scale bar, 100 µm. **d**, Table of engraftment efficiencies for HIO and SI CCS. Fisher’s exact test performed. * indicates significant difference between groups. *P* = 7.4 × 10^−7^ for kidney capsule HIO engraftment rates at d14 vs d28; *P* = 0.0014 for mesentery HIO engraftment rates at d14 vs d28; *P* = 9.25 × 10^−7^ for d14 mesentery HIO engraftment rate vs d14 mesentery SI CCS engraftment rate. **e**, Representative gross images of HIO and SI CCS at the time of collection (10 weeks post transplantation). **f**, Violin plot of HIO (*n* = 6) and SI CCS (*n* = 9) graft sizes. Two-tailed Mann–Whitney test performed. **g**, Representative images of H&E-stained sections of conventionally generated HIOs and SI CCS 10 weeks after transplantation. Scale bar, 100 µm. *n* ≥ 4 for histological staining. Schematic in **a** created in BioRender; Poling, H. https://biorender.com/s6t7j9e (2026). Source data

To impart a defined geometry on intestinal organoids, a specialized scaffolding tray was designed to culture mid-hindgut spheroids, and introduced into the traditional workflow (Fig. 1a). The scaffolding tray was made by 3D-printing a mould with longitudinal confinement lanes to culture a critical spheroid mass (Supplementary Fig. 1a). Biocompatible polydimethylsiloxane (PDMS) was then cured within the mould and removed for use (Supplementary Fig. 1b). This approach, termed the CCS, restricted spheroid growth within a defined space while enhancing scalability. Typically, a single spheroid forms a single HIO, but here, ~4,000 spheroids from spontaneous morphogenesis were used (Supplementary Fig. 1c). They began as discrete spheroids at d0 that developed into a unified construct by d5 (Fig. 1b). By d6, these structures were manually removed and re-plated in Matrigel for continued culture until d14 (Supplementary Fig. 1d). When comparing small intestinal CCS (SI CCS) to conventional HIOs at d6 and d14, the geometry was maintained, and both simple columnar epithelium and supporting mesenchymal populations persisted (Fig. 1c).

### Reduction in time to engraftment and enhanced morphometric features

**Extended Data Fig. 1:**
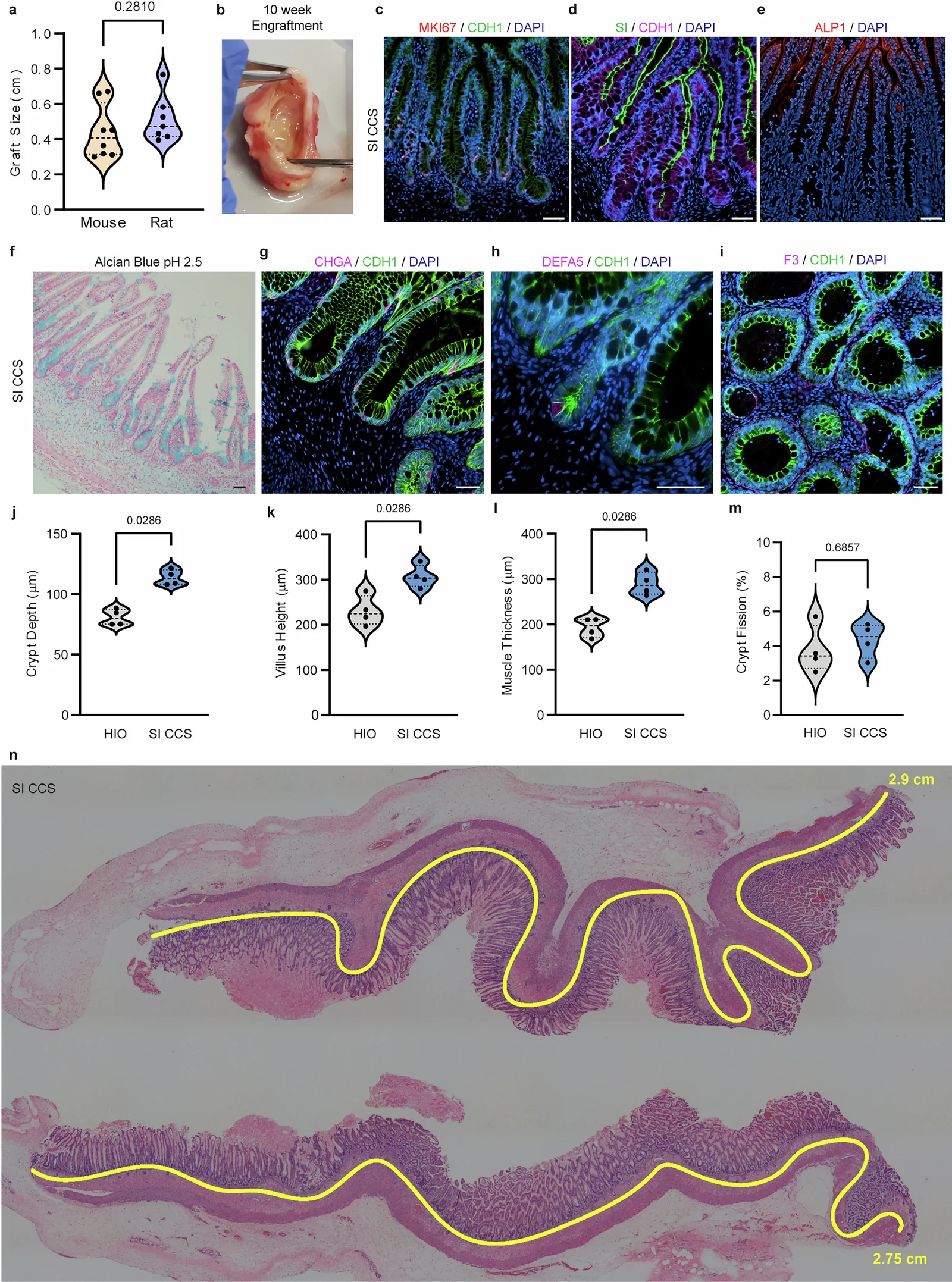
Intestinal morphology is enhanced in SI CCS. **a**, Violin plot of transplanted HIO size with mouse and rat as hosts. **b**, Photograph demonstrating the SI CCS central lumen after 10 weeks of engraftment. **c**, Representative staining for proliferation (MKI67, red) and epithelium (CDH1, green) in SI CCS. **d**, Representative staining for a brush border enzyme (SI, red) and epithelium (CDH1, green) in SI CCS. **e**, Representative image of active alkaline phosphatase activity in (ALP1, red) in SI CCS. **f**, Representative image of Alcian Blue pH 2.5 staining for mucins (blue) in SI CCS. **g**, Representative staining for enteroendocrine cells (CHGA, red) and epithelium (CDH1, green) in SI CCS. **h**, Representative staining for Paneth cells (DEFA5, red) and epithelium (CDH1, green) in SI CCS. **i**, Representative staining for telocytes (F3, red) and epithelium (CDH1, green) in SI CCS. **j-m**, Violin plots of morphometric quantifications of crypt depth (**j**), villus height (**k**), muscle thickness (**l**), and crypt fission (**m**) in transplanted HIOs and SI CCS. **n**, Tile scan of hematoxylin and eosin stained sections of SI CCS with yellow line highlighting continuous lengths of epithelium in sample. n = 3 for all staining panels. Scale bars in **c**-**i** = 50 µm. Mann-Whitney test performed on data in **a**, **j**, **k**, **l** and **m**. Source data

After d14 in culture, SI CCS structures displayed robustness in size and cellular integrity, suggesting sufficient maturation for transplantation. This contrasts with previous findings requiring HIOs to be cultured for at least 28 d before transplantation into the kidney capsule or mesentery of immunocompromised mice (Fig. 1d and Supplementary Fig. 1e)^14^. When transplanted at 14 d, SI CCS achieved 100% engraftment (Fig. 1d); however, the mouse’s abdominal space constrained growth, necessitating early tissue collection. To overcome this, we used an immunocompromised rat model (*Rag1*- and *Il2rg*-deficient rats, RRG) for all CCS transplants^17^. To assess whether host size influenced organoid growth potential, d28 HIOs were transplanted into mice and rats. Upon collection, no significant size differences were observed, excluding host size as a variable. Previous studies also demonstrated that transplantation site did not affect outcomes (Extended Data Fig. 1a)^15^. On the basis of these findings, we refined animal use, transplanting conventional organoids into mice and CCS organoids into rats.

After 10 weeks, SI CCS and HIO grafts were collected (Fig. 1e). SI CCS grafts were significantly larger than HIO grafts, reaching widths of 8 cm with large central lumens (Fig. 1f and Extended Data Fig. 1b). Histological analysis confirmed a stereotypical intestinal phenotype (Fig. 1g), with appropriate proliferative zonation as demonstrated by marker of proliferation Ki-67 (MKI67) protein staining (Extended Data Fig. 1c). Digestive enzyme sucrase isomaltase (SI) and functional alkaline phosphatase (ALP1) were demonstrated via immunofluorescence (Extended Data Fig. 1d,e). Secretory epithelial cell types were also identified and observed through staining for Alcian blue for mucin-producing goblet cells, chromogranin A (CHGA) protein marking enteroendocrine cells, and antimicrobial peptide alpha defensin 5 (DEFA5) labelling Paneth cells (Extended Data Fig. 1f–h). Subepithelial telocytes, important supporting cells of the intestinal epithelium, were also observed and localized adjacent to the epithelium^16^ (Extended Data Fig. 1i). Morphometric analysis revealed that SI CCS had increased crypt depth, villus height and muscle thickness compared to HIOs (Extended Data Fig. 1j–l). No differences in crypt fission were observed, probably due to size plateauing under homeostatic conditions (Extended Data Fig. 1m). Notably, SI CCS exhibited vast stretches of continuous epithelium spanning several centimetres (Extended Data Fig. 1n), demonstrating enhanced epithelial architecture and structural maturity.

### Co-emergence of a de novo enteric nervous system

**Fig. 2:**
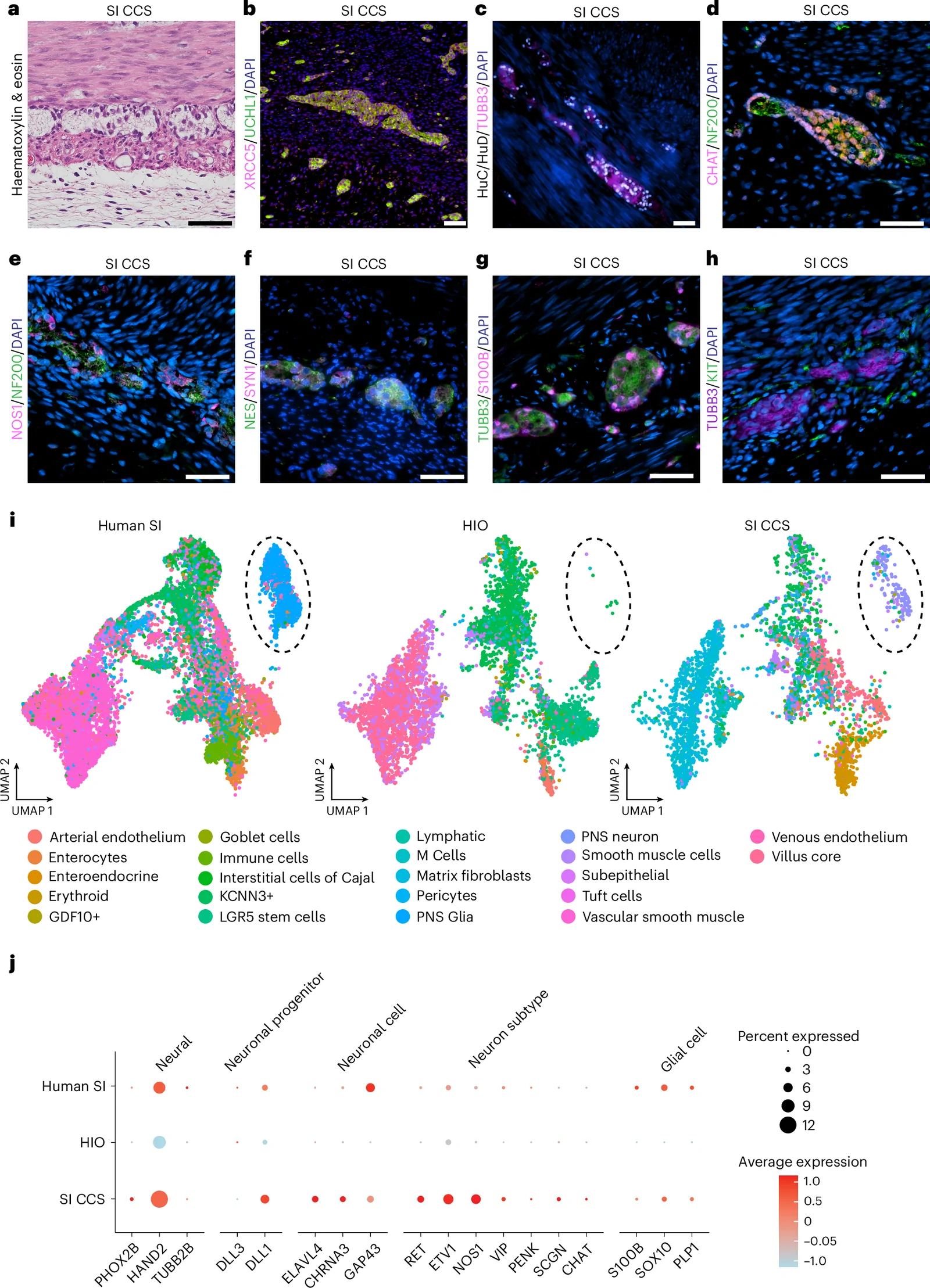
A robust ENS co-developed within SI CCS. **a**, Representative image of H&E-stained section of SI CCS 10 weeks after transplantation. **b**, Immunofluorescence for a human-specific marker (XRCC5, pink) and a pan-neuronal marker (UCHL1, green) in SI CCS. **c**, Immunofluorescence for two pan-neuronal markers (TUBB3, green; and HuC/HuD, white) in SI CCS. **d**, Immunofluorescence for neurofilaments (NF200, green) and a cholinergic neuronal marker (CHAT, pink) in SI CCS. **e**, Immunofluorescence for neurofilaments (NF200, green) and a nitrergic neuronal marker (NOS1, pink) in SI CCS. **f**, Immunofluorescence for intermediate neurofilaments (NES, green) and synaptic vesicles (SYN1, pink) in SI CCS. **g**, Immunofluorescence for glia (S100B, pink) and neurons (TUBB3, green) in SI CCS. **h**, Immunofluorescence for neurons (TUBB3, purple) and ICCs (KIT, green) in SI CCS. **i**, UMAPs of snRNA-seq datasets generated from the human small intestine, transplanted HIO and transplanted SI CCS with query transferred labels from curated reference atlas. Dashed oval indicates the localization of neuroglial cell clusters. **j**, Dot plot of transcription levels from snRNA-seq datasets of genes related to the ENS. All scale bars, 50 µm. *n* ≥ 4 for all histological and immunofluorescence staining.

Looking beyond the epithelium, SI CCS exhibited a robust network of myenteric plexuses, appropriately localized between circular and longitudinal muscle layers (Fig. 2a). Immunofluorescence staining confirmed neuronal presence using pan-neuronal protein marker Ubiquitin C-Terminal Hydrolase L1 (UCHL1, aka PGP9.5), and a human cellular protein marker, X-Ray Repair Cross Complementing 5 (XRCC5, aka KU80). This suggests (1) the presence of neurons and (2) that their origin was not due to host neuronal ingrowth (Fig. 2b)^18^. As further confirmation, staining for additional pan-neuronal protein markers Tubulin Beta 3 Class III (TUBB3) and ELAV Like RNA Binding Proteins 3 and 4 (ELAV3/4, aka HUC/D) were also observed in SI CCS (Fig. 2c). Excitatory cholinergic and inhibitory nitrergic neuronal subtypes were also present in the SI CCS^19^. Cholinergic neurons were demonstrated by co-staining for Choline O-Acetyltransferase (CHAT) protein and Neurofilament Protein (NF200), while nitrergic neurons were shown by co-staining for Nitric Oxide Synthase 1 (NOS1) and NF200 proteins (Fig. 2d,e)^20^. Synaptic vesicles (Synapsin1, SYN1) and an intermediate filament protein nestin (NES) were also observed throughout the muscularis of SI CCS (Fig. 2f)^20,21^. We also confirmed the presence of glial cells using S100 Calcium Binding Protein B (S100B) as a protein marker, which were closely associated with neurons (Fig. 2g). Because interstitial cells of Cajal (ICCs) localize near neuronal bundles and serve as the pacemaker of the gut, we investigated their presence via protein expression using KIT Proto-Oncogene, Receptor Tyrosine Kinase (KIT) and found that they were in close association with TUBB3+ cell bundles (Fig. 2h).

**Extended Data Fig. 2:**
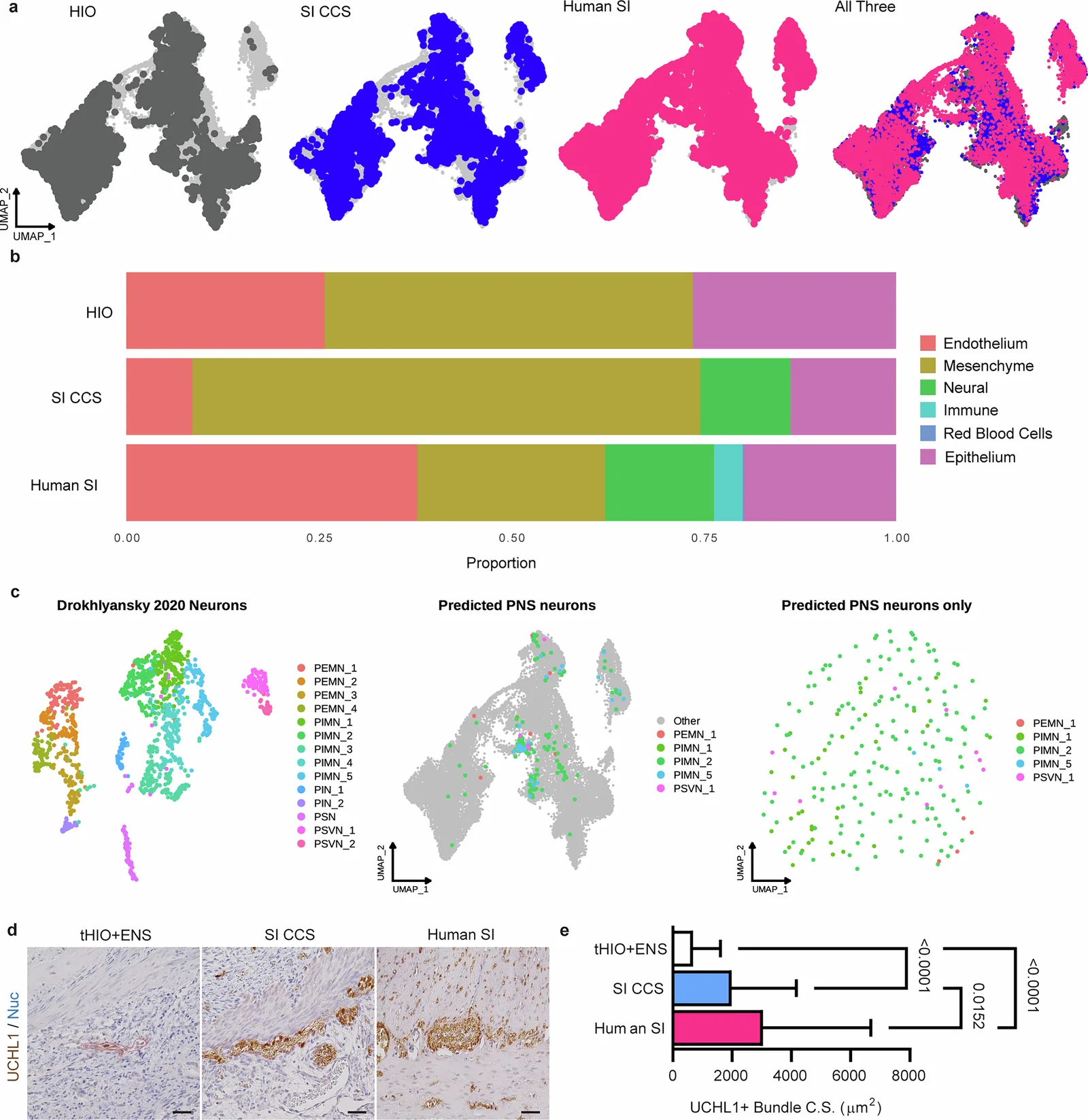
Neural cell populations are well represented in SI CCS. **a**, UMAPs of transplanted HIO, SI CCS and Human SI data sets projected individually onto the reference and combined. **b**, Bar graph representation of cell type proportions in transplanted HIO, SI CCS and Human SI. **c**, UMAPs of SI CCS neural cells projected neuronal classes using the Drokhlyansky dataset as reference. **d**, Representative staining for neurons (UCHL1, brown) in transplanted HIO + ENS, SI CCSs, and human small intestine. Scale bars = 100 µm. **e**, Bar graph (mean+SD) of measured cross sectional UCHL1+ bundle sizes from samples in panel d. Bundle sizes in SI CCS were significantly larger than those in transplanted HIO + ENS. ANOVA with Tukey’s post hoc analysis performed. n = 3 per group.

As an orthogonal method to confirm ENS presence, single nuclear isolations were performed on the human small intestine, transplanted HIOs and transplanted SI CCS for single nuclear RNA sequencing (snRNA-seq). A reference atlas was curated from three publicly available single-cell RNA sequencing (scRNA-seq) datasets, including STAR-FINDer^22^, the Gut Cell Survey^23^ and the Endodermal Organ Cell Atlas^24^. Uniform Manifold Approximation and Projection (UMAP) analysis confirmed successful integration of the datasets and reference (Extended Data Fig. 2a). Label transfer analysis validated our protocol’s ability to isolate ENS cell types, as neurons and glia were identified in human samples. Transplanted HIOs lacked these populations, whereas SI CCS contained ENS-related populations, further confirming neuroglial identity (Fig. 2i). We also defined the proportions of umbrella cell types (endothelium, mesenchyme, neural, immune, red blood cells and epithelium) across sample types. We observed an increase in both mesenchymal and neuronal populations in SI CCS compared to HIO (Extended Data Fig. 2b).

To specifically investigate the transcription levels of ENS-related genes, a dot plot was generated from the three datasets (Fig. 2j). The average expression for genes related to neuronal progenitors, neuronal cells and subtypes, and glial cells was more robust in SI CCS than in HIO which displayed little to no expression across the gene panel. The presence of neuronal subtype-related gene expression led us to make neuronal subtype predictions utilizing previously published datasets (Extended Data Fig. 2c)^25^. Using this approach, putative excitatory motor neurons, putative inhibitory motor neurons and putative secromotor/vasodilator neurons were predicted within the SI CCS dataset, further suggesting neuronal heterogeneity.

Our group previously generated HIOs with an ENS by adding exogenous neural crest cells to spheroids (HIO + ENS assembloids)^26^. To compare the ENS generated by both methodologies, we examined UCHL1+ myenteric plexuses in transplanted HIO + ENS, SI CCS and human intestinal samples via protein expression. The plexus size was significantly larger in SI CCS, suggesting that co-development within CCS enhances innervation more effectively than assembling ENS components separately (Extended Data Fig. 2d,e).

### Neuromuscular function in SI CCS

**Fig. 3:**
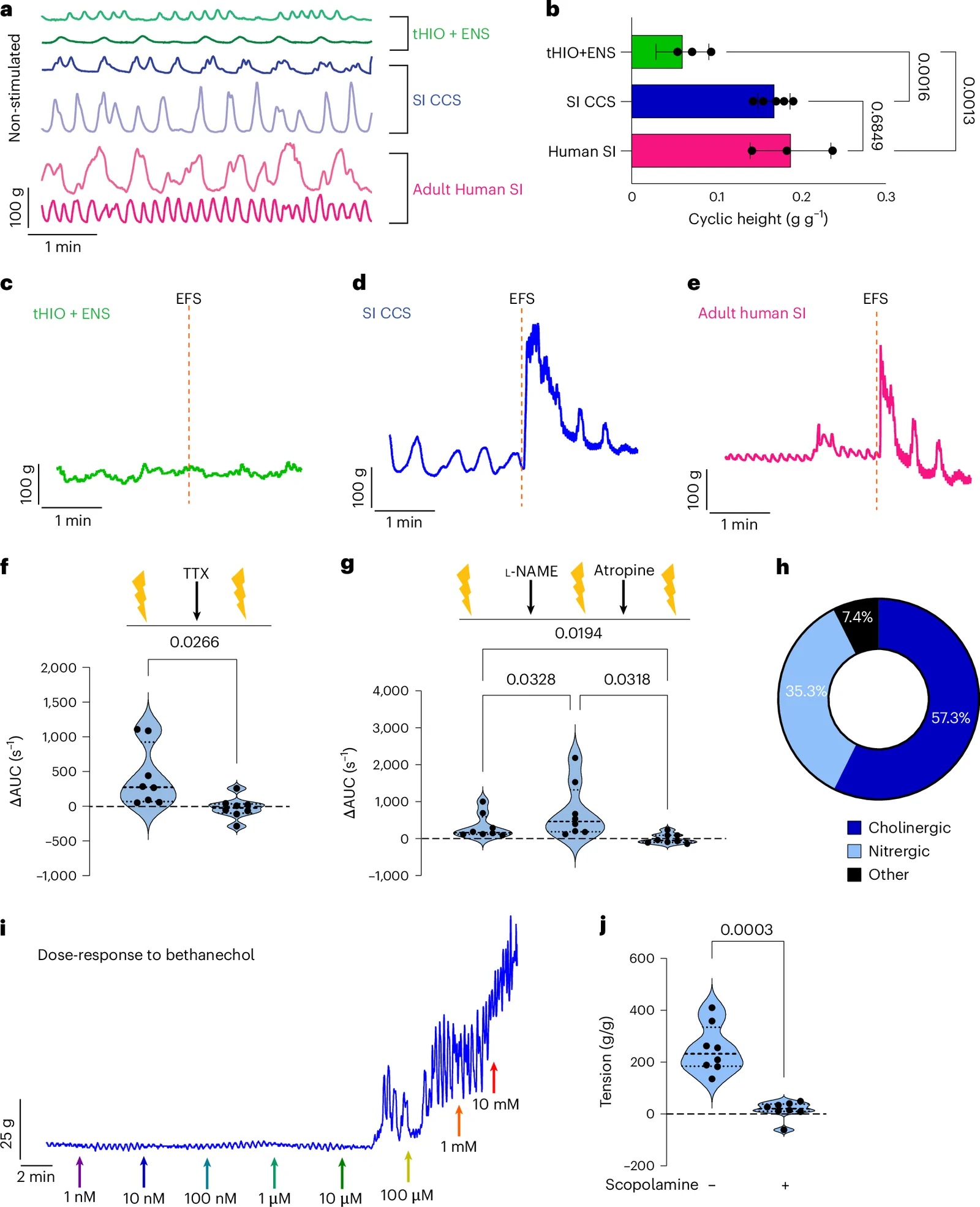
SI CCS contained a functional ENS that regulates smooth muscle contractility. **a**, Isometric force contractions of muscle strips from tHIO + ENS, SI CCS and adult human SI after an equilibration period. Two representative tracings per condition, from independent experiments, are represented. **b**, Bar graph (mean ± s.d.) of the average cyclic height of contractile tracings from tHIO + ENS (*n* = 4), SI CCS (*n* = 5) and adult human SI (*n* = 3). SI CCS and adult human SI values demonstrated no significant difference between groups. Two-tailed Mann–Whitney test performed. **c**, Contractile activity tracing in tHIO + ENS with EFS stimulation (dashed line indicates the administration time). **d**, Contractile activity tracing in SI CCS with EFS stimulation (dashed line indicates the administration time). **e**, Contractile activity tracing in adult human SI with EFS stimulation (dashed line indicates the administration time). **f**, TTX inhibition of ENS activity in SI CCS. Violin plot of the change in the area under the curve (AUC) before and after TTX administration; after TTX, neuronal response was significantly reduced. Two-tailed Mann–Whitney test performed. **g**, Nitrergic and cholinergic inhibition of ENS activation. Violin plot of the change in the AUC following control EFS stimulation, followed by L-NAME treatment, another EFS stimulation, followed by atropine treatment and a final EFS stimulation in SI CCS. EFS responses were significantly different from one another. Friedman test with Dunn’s multiple comparisons performed. **h**, Average calculated neuronal subtype functional contribution from experiments in **g**. **i**, Representative bethanechol dose-response tracing of contractile activity in a transplanted SI CCS; with dosage increases, tensile activity increased. Coloured arrows indicate the timing of logarithmic bethanechol doses. **j**, Calculated maximal and minimal tissue tension of SI CCS; scopolamine was used to induce muscle relaxation. Two-tailed Mann–Whitney test performed. *n* = 8 for organ bath experiments and in plots (**f**,**g**,**j**). All reported data were normalized to tissue mass. Source data

Organ bath chamber assays were conducted to assess neuromuscular function in SI CCS. Continuous monitoring of contractile activity revealed rhythmic isometric force contractions after equilibration (Fig. 3a). To contextualize this non-stimulated activity, transplanted HIO + ENS and human small intestinal samples were also analysed. Contractile amplitude was significantly greater in SI CCS than in HIO + ENS and comparable to human intestine (Fig. 3b). Selective nerve excitation through electric field stimulation (EFS) was then performed on all three sample types (Fig. 3c–e). HIO + ENS showed minimal contractile activity, consistent with previous findings^26^, while SI CCS and human intestine exhibited strong contractile responses, confirming successful nerve excitation. ENS dependence was validated by tetrodotoxin (TTX) application, which significantly reduced contractile responses upon repeated EFS (Fig. 3f). To investigate the functional contributions of cholinergic and nitrergic neurons, which were demonstrated through protein expression, nitric oxide synthetase (nNOS)-expressing neurons were inhibited with NG-nitro-l-arginine methyl ester (L-NAME), followed by cholinergic inhibition using atropine^26^. Contractile activity associated with EFS was measured before and after each treatment (Fig. 3g). These data demonstrated that SI CCS contractions involved both nitrergic and cholinergic neuronal activity, with the cholinergic component being more dominant (Fig. 3h). These functional data represent a more holistic view of the ENS including various cell type interactions beyond those captured in the snRNA-seq datasets; however, both functional and snRNA-seq data indicate the presence of nitrergic and cholinergic neuron populations.

Smooth muscle function, independent of ENS influence, was assessed by administering bethanechol, a muscarinic receptor agonist. Contractile force increased in a dose-dependent manner (Fig. 3i), confirming functional smooth muscle formation in SI CCS. These effects were reversible, as smooth muscle relaxation was induced with scopolamine, a muscarinic antagonist (Fig. 3j). Together, these results demonstrate that SI CCS successfully generates functional smooth muscle, which operates independently and in conjunction with ENS stimulation.

### The CCS promotes in vitro ENS development

Previously, generating organoid tissues with an ENS required adding exogenous neural crest cells, differentiated separately and incorporated with spheroids^11,26,27^. Surprisingly, SI CCS developed a functional ENS without this method. To gain insight into the emergence of the ENS in SI CCS, transcriptomic analysis was performed in vitro at d14, first between HIO and SI CCS. When examining the heat map of the two groups, d14 HIOs and d14 SI CCS segregated and presented with transcriptional differences (Supplementary Fig. 2a). Differentially expressed genes in SI CCS highlighted enriched gene ontologies related to ENS development (Supplementary Fig. 2b). The top five biological processes and cellular components were all linked to early ENS formation, suggesting that CCS promotes endogenous ENS development within the organoids.

### The in vitro SI CCS ENS represents an early developmental state

**Fig. 4:**
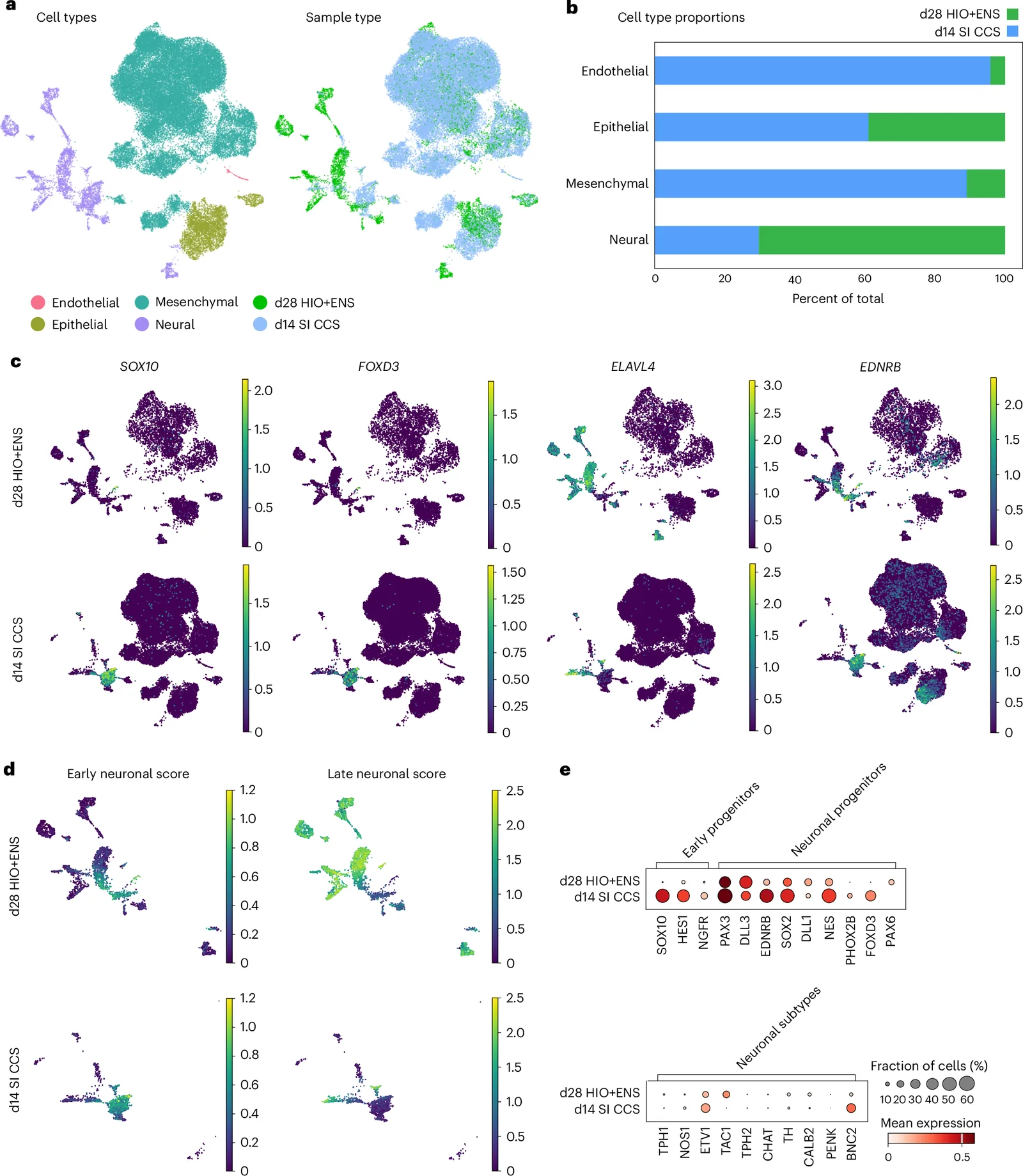
Transcriptional profiling reveals an enrichment in early ENS progenitors within SI CCS pre-transplantation. **a**, UMAPs of in vitro d28 HIO + ENS and d14 SI CCS scRNA-seq colour coded by cell type identity (grouped as endothelial, epithelial, mesenchymal and neural) and according to sample type. **b**, Bar graph of cell type proportions in d28 HIO + ENS and d14 SI CCS. **c**, Feature plots highlighting transcription of genes SOX10, FOXD3, ELAVL4 and EDNRB in d28 HIO + ENS and d14 SI CCS. (Color scales indicates normalized expression of gene levels across cells in UMAP space). **d**, UMAPs of the isolated neural cluster in d28 HIO + ENS and d14 SI CCS indicating early and late neuronal scores across each sample type. d28 HIO + ENS demonstrate more widespread late neuronal scores, while d14 SI CCS demonstrate early neuronal scores. **e**, Dot plot of transcription levels from scRNA-seq datasets of genes related to the early progenitors, neuronal progenitors and neuronal subtypes.

Next, we compared the de novo ENS in SI CCS to that in HIO + ENS. EFS failed to induce responses in engrafted HIO + ENS, but produced robust responses in SI CCS, indicating functional differences. To understand factors that may have contributed to this functional difference related to engraftment, scRNA-seq was performed on d28 HIO + ENS and d14 SI CCS, their respective stages for transplantation. First, a harmonized UMAP was generated and four umbrella cell types (endothelial, epithelial, mesenchymal and neural) were identified (Fig. 4a). Then, the integration of the two sample types was explored through another UMAP, colour coded by group (Fig. 4a). A bar graph was generated using the four umbrella cell types to examine differences in cell type proportions (Fig. 4b). Associated raw event counts were also calculated (Supplementary Fig. 2c). Interestingly, the d14 SI CCS had a smaller neural proportion than the d28 HIO + ENS. However, for the other categories (endothelial, epithelial and mesenchymal), d14 SI CCS demonstrated a larger proportion than d28 HIO + ENS. This may be an artefact of dissociation, or because the neural population was exogenously provided within the HIO + ENS. Irrespective of the cause, we wanted to interrogate further the neural populations captured in both groups. As a first line assessment, feature plots visualized key ENS-related genes (Fig. 4c). *SRY-Box Transcription Factor 10* (*SOX10*), which encodes a transcription factor that helps maintain neural cells in an undifferentiated proliferative state, was rarely observed within the d28 HIO + ENS, but well represented within d14 SI CCS^28^. *Forkhead Box D3* (*FOXD3*), which encodes a transcription factor expressed in early neural crest cells, pivotal in maintaining their multipotency, demonstrated a similar expression pattern^29^. *ELAV Like RNA Binding Protein 4* (*ELAVL4*), which encodes a protein marker for cells that have committed to a neuronal fate, was more broadly expressed within the neuronal cluster of d28 HIO + ENS than in d14 SI CCS^30^. *Endothelin Receptor Type B* (*EDNRB*), which encodes a protein that plays an early role in neural migration and the prevention of premature differentiation, seemed represented to a similar level in the neural clusters of both d28 HIO + ENS and d14 SI CCS^31,32^.

To further assess neuronal maturity, early and late neuronal scores were established on the basis of well-characterized ENS differentiation markers. d28 HIO + ENS exhibited high late neuronal scores, whereas d14 SI CCS demonstrated high early neuronal scores (Fig. 4d). Neural subpopulations were then stratified using the Fetal Gut Cell Atlas enteric dataset (Supplementary Fig. 2d). Best-match predictions revealed that early progenitor subclusters, including enteric neural crest cells (ENCC)/glia progenitors, cycling ENCC/glia, neuroblasts and cycling neuroblasts, accounted for ~40% of the neural cluster in d28 HIO + ENS but over 80% in d14 SI CCS (Supplementary Fig. 2e). Dot plots confirmed the enrichment of early progenitor markers in d14 SI CCS compared to d28 HIO + ENS (Fig. 4e). These findings indicate that the ENS within SI CCS is at an earlier developmental stage, with a higher proportion of progenitors, potentially contributing to its enhanced functional capacity upon engraftment.

### Extending the CCS technique to generate additional gastrointestinal tissue types

Current gastrointestinal organoid protocols often result in poor engraftment and small size, limiting physiological studies and clinical applications^5–7,9,11^. Assembloid approaches, differentiation of key components separately before recombination, can mitigate these issues but also introduces new challenges related to cell ratios and timing^11,25,26^. To address this, we applied our CCS methodology to generate colonic and gastric tissue, streamlining organoid development and improving overall transplant potential.

**Fig. 5:**
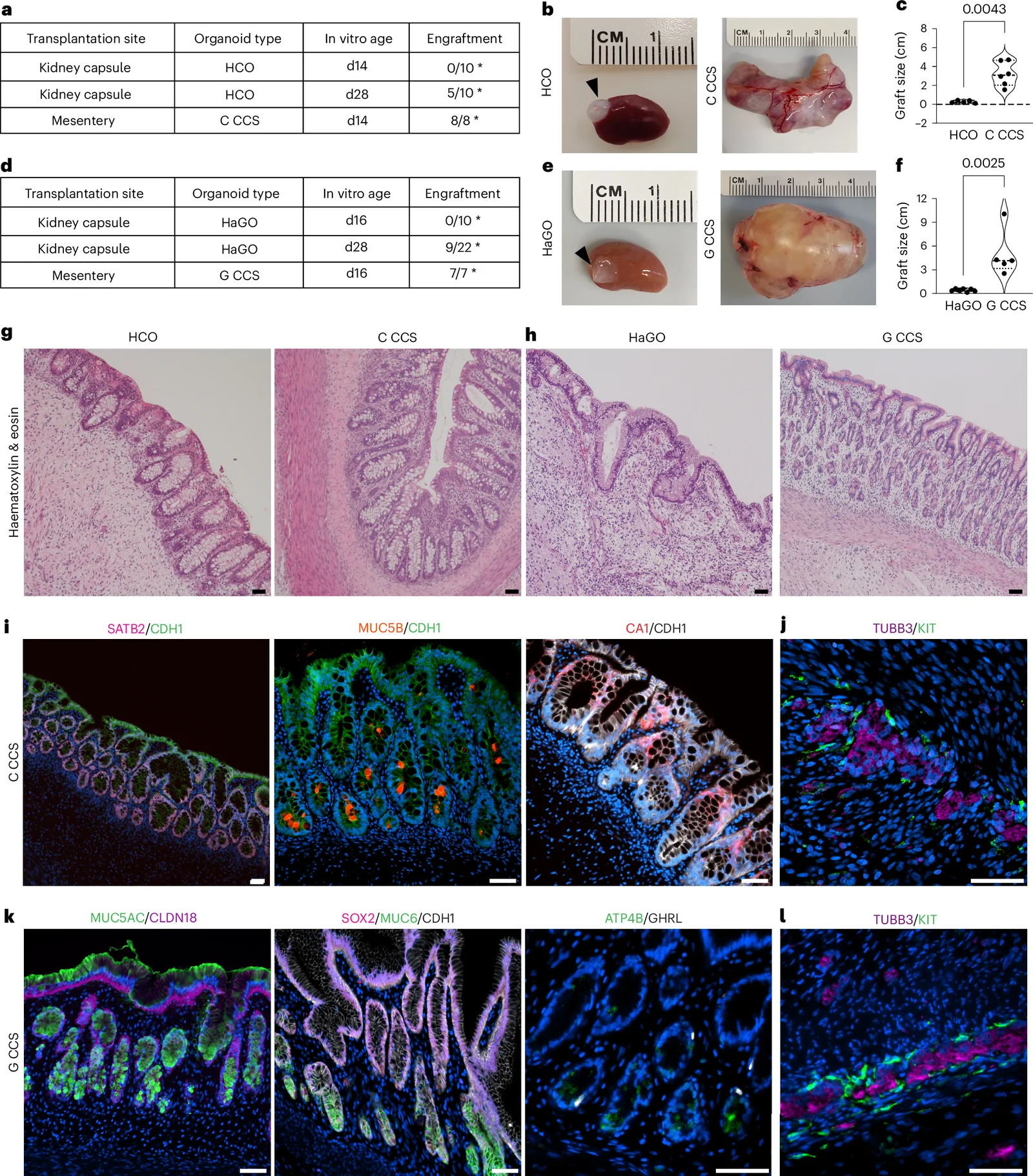
The scaffolding tray methodology successfully generated colonic and gastric tissue. **a**, Engraftment rates of conventionally generated HCOs and C CCS. Fisher’s exact test performed. * indicates significant difference between groups; *P* = 0.0325 for d14 vs d28 HCO engraftment rates; *P* = 2.29 × 10^−5^ for d14 HCO vs d14 C CCS engraftment rates; *P* = 0.036 for d28 HCO vs d14 C CCS engraftment rates. **b**, Representative gross images of HCO and C CCS at the time of collection (10 weeks post transplantation). **c**, Violin plot of HCO (*n* = 5) and C CCS (*n* = 6) graft sizes. Two-tailed Mann–Whitney test performed. **d**, Engraftment rates of conventionally generated HaGOs and G CCS. Fisher’s exact test performed. * indicates significant difference between groups; *P* = 0.0303 for d16 vs d28 HaGO engraftment rates; *P* = 5.14 × 10^−5^ for d16 HaGO vs d14 G CCS engraftment rates; *P* = 0.0084 for d28 HaGO vs d14 G CCS engraftment rates. **e**, Representative gross images of HaGO and G CCS at the time of collection (10 weeks post transplantation). **f**, Violin plot of HaGO (*n* = 7) and G CCS (*n* = 5) graft sizes. Two-tailed Mann–Whitney test performed. **g**, Representative images of H&E-stained sections of conventionally generated HCO and C CCS 10 weeks after transplantation. **h**, Representative images of H&E-stained sections of conventionally generated HaGO and G CCS 10 weeks after transplantation. **i**, Representative immunofluorescence staining for distal and colonic markers in transplanted C CCS: distal transcription factor (SATB2, pink) and epithelium (CDH1, green), a colonic mucin (MUC5B, orange) and epithelium (CDH1, green), and a colonic epithelial enzyme (CA1, red) and epithelium (CDH1, white). **j**, Representative immunofluorescence for neurons (TUBB3, purple) and ICCs (KIT, green) in transplanted C CCS. **k**, Representative immunofluorescence staining for gastric markers in transplanted G CCS: gastric mucin (MUC5AC, green) and gastric epithelium (CLDN18, purple), a transcription factor (SOX2, purple), an antral gastric mucin (MUC6, green) and epithelium (CDH1, white), and a parietal cell transporter (ATP4B, green) and gastric hormone ghrelin (GHRL, white). **l**, Representative immunofluorescence for neurons (TUBB3, purple) and ICCs (KIT, green) in transplanted G CCS. **g**–**l**, Scale bars, 50 µm. *n* ≥ 4 for all histological and immunofluorescence staining. Arrowheads in **b** and **e** denote sites of engraftment. Source data

We explored whether the CCS methodology could generate colonic and gastric tissue, given that those protocols also rely on spheroid morphogenesis from monolayers. Using a previously published protocol, we introduced transient bone morphogenetic protein (BMP) activation for organoid posteriorization^7^. Similar to SI CCS, colonic CCS (C CCS) successfully engrafted at d14, whereas conventionally generated HCOs required an additional 14 d of maturation (Fig. 5a). Engraftment efficiency was significantly improved with the scaffolding tray (Fig. 5a). After 10 weeks, HCOs and C CCS were collected, with C CCS reaching widths of ~6 cm (Fig. 5b,c). Foregut spheroids^6,11^ placed in scaffolding trays also improved antral gastric tissue (G CCS) engraftment and size (Fig. 5d–f).

Histologically, C CCS displayed a characteristic colonic phenotype lacking villi with a well-defined muscularis, which was largely absent in transplanted HCOs (Fig. 5g). G CCS exhibited organized pits and glands; however, a more rudimentary structure was found in conventional HaGO (Fig. 5h). To further support the tissue type identity of C CCS and G CCS organoids, immunofluorescence for protein expression of regional markers was performed. C CCS were found to express proteins SATB Homeobox 2 (SATB2, a marker of distal gut epithelium^7^), Mucin 5B (MUC5B, a gel-forming mucin expressed by a subset of colonic goblet cells^33^), as well as a colonic enzyme Carbonic Anhydrase 1 (CA1)^34^ supporting the idea that colonic patterning was successful (Fig. 5i). Next, pan-neuronal staining was performed to determine whether neuronal co-development persisted with C CCS generation. Interestingly, nerves were again observed in close association with ICCs (Fig. 5j). G CCS were found to express proteins Mucins 5AC and 6 ((MUC5AC, MUC6)^33^, gel-forming mucins expressed by some gastric goblet cells), Claudin 18 ((CLDN18)^35^, a gastric transmembrane protein) and SRY-Box Transcription Factor 2 ((SOX2)^36^, expressed during stomach development) (Fig. 5k). Parietal cells, marked by ATPase H + /K+ Transporting Subunit Beta (ATP4B) protein expression^37^, as well as the gastric hormone ghrelin (GHRL)^38^, were also observed within G CCS samples, supporting their successful gastric patterning (Fig. 5k). Then, neuronal co-development was demonstrated through pan-neuronal staining in G CCS samples, establishing that CCS fostered the co-emergence of an ENS in all three contexts investigated (Fig. 5j).

**Extended Data Fig. 3:**
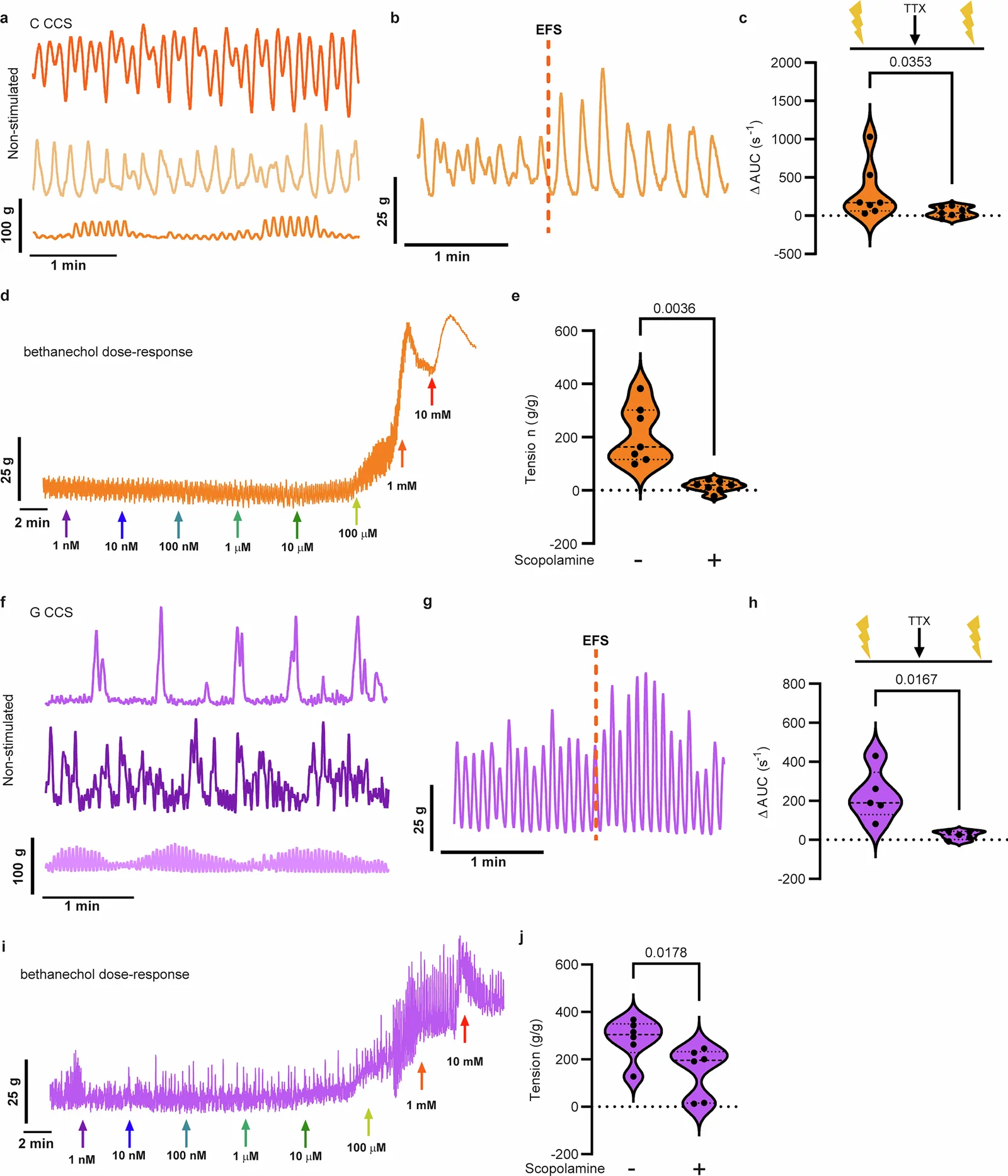
C CCS and G CCS recapitulates functional human colonic and gastric tissues. **a**, Contractile activity tracings in C CCS under homeostatic conditions. **b**, Contractile tracing in C CCS with EFS stimulation (dashed line indicates time of administration). **c**, TTX inhibition of ENS activity in C CCS. Violin plot of the change in the area under the curve before and after TTX. **d**, Representative bethanechol dose-response tracing of contractile activity in a transplanted C CCS. Colored arrows indicate the timing of logarithmic bethanechol doses. **e**, Calculated maximal and minimal tissue tension of C CCS; scopolamine was used to induce muscle relaxation. **f**, Contractile activity tracings in G CCS under homeostatic conditions. **g**, Contractile tracing in G CCS with EFS stimulation (dashed line indicates time of administration). **h**, TTX inhibition of ENS activity in G CCS. Violin plot of the change in the area under the curve before and after TTX. **i**, Representative bethanechol dose-response tracing of contractile activity in a transplanted G CCS. Colored arrows indicate the timing of logarithmic bethanechol doses. **j**, Calculated maximal and minimal tissue tension of G CCS; scopolamine was used to induce muscle relaxation. n = 7 C CCS and n = 6 G CCS for organ bath experiments. All data reported was normalized to tissue mass. Two-tailed paired Student T Test performed for data in **c**, **e**, **h**, and **j**. Source data

Next, we investigated whether the ENS in the C CCS and G CCS tissues was functional through organ bath chamber assays similar to those performed on SI CCS. First, when interrogating the C CCS, rhythmic isometric force contractions were observed after a period of equilibration (Extended Data Fig. 3a). Next, we investigated whether mature neuromuscular coupling was also present through selective nerve excitation. EFS pulses were administered, and contractile activity was observed, indicating that the ENS regulated smooth muscle (Extended Data Fig. 3b). To confirm that this phenomenon was ENS dependent, neuronal activity was inhibited with TTX, yielding a significantly reduced contractile response after another EFS application (Extended Data Fig. 3c). Smooth muscle function was interrogated by the administration of bethanechol followed by scopolamine, in which contraction and relaxation were successfully induced (Extended Data Fig. 3d,e). These assessments were repeated on G CCS, yielding similar results (Extended Data Fig. 3f–j). Thus, the CCS-based methodology successfully generates colonic and gastric tissue, both featuring a functional ENS.

### Proof of concept for the clinical applicability of SI CCS

Exposing intestinal organoids to luminal content in vivo remains a challenge. Our group previously developed the ‘tie in’ procedure, where an engrafted organoid was anastomosed to the host’s bowel, enabling the study of intestinal adaptation from a sterile to a fed state with bacterial colonization and nutrient exposure (Supplementary Fig. 3a)^39^. However, high mortality limited long-term studies (Supplementary Fig. 3b). Nevertheless, by 5 d we observed the establishment of continuous epithelia between the host and transplanted organoid as demonstrated through immunofluorescence staining for proteins CDH1 and XRCC5 (Supplementary Fig. 3c). The stereotypical intestinal proliferative zonation of the host and organoid remained intact after the procedure, as observed by immunofluorescence staining for MKI67 protein (Supplementary Fig. 3c). In addition, histological staining for bacteria was performed and multiple bacterial phenotypes were observed near the organoid’s epithelium (Supplementary Fig. 3d). Organoid luminal contents were cultured, confirming bacterial outgrowth exclusively in the ‘tie in’ group (Supplementary Fig. 3e). In response to luminal exposure, increased mucin production was observed histologically at d5, with elevated acidic and strongly sulfated mucosubstances (Supplementary Fig. 3f). By d12, mucin levels declined but remained higher than in controls.

**Fig. 6:**
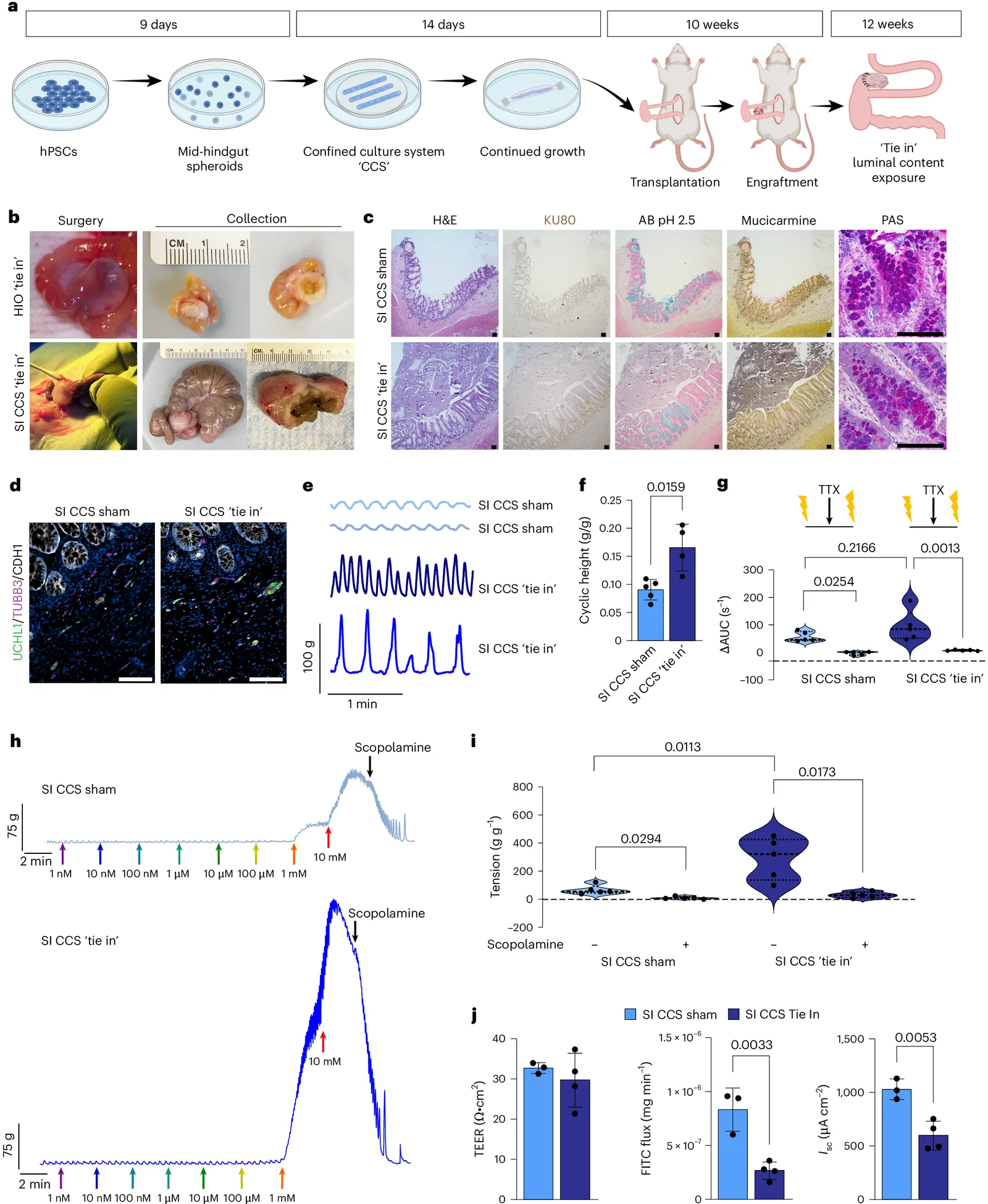
SI CCS robustly modelled adaptation to the fed intestinal state. **a**, Experimental schematic describing the generation and transplantation of SI CCS, engraftment and secondary ‘tie in’’ procedure to expose the engrafted tissue to host luminal content. **b**, Representative photographs of mouse HIO ‘tie in’ and rat SI CCS ‘tie in’ at the time of surgery and collection. **c**, Representative staining panels of mucin profiles on serial sections of SI CCS sham and SI CCS ‘tie in’. **d**, Representative immunofluorescence images of SI CCS sham and SI CCS ‘tie in’ stained for neurons (UCHL1, green; TUBB3, purple) and epithelium (CDH1, white). **e**, Isometric force contractions of muscle strips from SI CCS sham and SI CCS ‘tie in’ after an equilibration period. Two representative tracings per condition, from independent experiments, are represented. **f**, Bar graph (mean ± s.d.) of the average cyclic height of contractile tracings from SI CCS sham (*n* = 5) and SI CCS ‘tie in’ (*n* = 4). Two-tailed Mann–Whitney test performed. **g**, TTX inhibition of ENS activity in SI CCS sham and SI CCS ‘tie in’ (*n* = 5 per group). Violin plot of the change in the AUC before and after TTX administration; after TTX, neuronal response was significantly reduced for both groups. Analysis of variance (ANOVA) with Tukey’s post hoc analysis was performed. **h**, Contractile activity tracings in SI CCS sham and SI CCS ‘tie in’ in response to logarithmic doses of bethanechol, followed by scopolamine administration. Arrows indicate administration timings. **i**, Calculated maximal and minimal tissue tension of SI CCS sham and SI CCS ‘tie in’ (*n* = 5 per group). ANOVA with Tukey’s post hoc analysis was performed. **j**, Bar graphs (mean ± s.d.) of functional epithelial characteristics in SI CCS sham (*n* = 3) and SI CCS ‘tie in’ (*n* = 4) as measured in a Ussing chamber assay. From left to right: transepithelial resistance, paracellular permeability and short-circuit current. Two-tailed unpaired Student’s *t*-tests were performed. All scale bars, 100 µm. Schematic in **a** created in BioRender; Poling, H. https://biorender.com/91sfgeo (2026). Source data

Transepithelial resistance (TEER) and paracellular permeability were also evaluated through electrophysiology at the d5 timepoint, and were found to be similar between groups. However, short-circuit current (*I*_sc_), a measure of active ion exchange, was reduced in ‘tie ins’, suggesting an increase in negative ion trafficking (Supplementary Fig. 3g). Despite these insights, the model had substantial challenges, including the need for highly skilled surgeons, strict dietary control and small organoid size, limiting downstream analysis. To overcome these, we adapted the ‘tie in’ procedure using SI CCS (Fig. 6a).

The CCS model in rats significantly reduced mortality (Supplementary Fig. 3c) and allowed for the dietary transition back to chow, enabling extended 12-week studies. Larger organoid tissue facilitated more comprehensive histological and electrophysiological analyses, while improved survival and engraftment refined animal usage (Fig. 6b). For these procedures, part of the SI CCS was transplanted into the host’s mesentery to facilitate subsequent anastomosis to the caecum, chosen for its high microbial density and permissive anatomy. Similar to the mouse model, luminal contents filled the SI CCS ‘tie in’ at collection (Fig. 6b), and histology confirmed adaptive architecture with visible luminal contents (Fig. 6c). Mucin profiles in SI CCS ‘tie ins’ were homeostatic and heterogeneous, consistent with d12 HIO ‘tie ins’ (Fig. 6c). While a goblet cell rich epithelium was observed, the quantity of goblet cells per unit length of epithelium was decreased with the ‘tie in’ procedure (Supplementary Fig. 4a).

Next, we characterized functional maturation within the system. Interestingly, staining for pan-neuronal protein markers (TUBB3 and UCHL1) revealed continued ENS development within the SI CCS ‘tie in’ and sham (Fig. 6d). At 10 weeks, most of the neuronal structures were associated with the smooth muscle layers. However, at the 22-week timepoint, a more consistent incorporation was observed throughout the full thickness of the tissue, particularly in the submucosal space, in both groups. This indicated that the ENS development had not yet plateaued at the 10-week timepoint and that neuronal expansion occurred and was independent of the ‘tie in’ procedure. During adolescence, substantial architectural remodelling and changes occur within the ENS, particularly in the submucosal regions. This correlates with the most notable changes observed in the SI CCS tissues between 10 and 22 weeks of transplantation^40^.

When examining contractile tracings of the SI CCS ‘tie in’ and sham under non-stimulated conditions, the SI CCS ‘tie in’ had significantly increased amplitudes compared to sham (Fig. 6e,f). EFS responses were comparable in both groups (Fig. 6g), with contractions significantly reduced after tetrodotoxin (TTX) treatment, confirming ENS dependence (Fig. 6g). These data suggest that luminal content exposure enhances ENS maturation beyond the effects of engraftment duration. Next, muscle tone was explored between the groups by administering logarithmic doses of bethanechol. During this experiment, live tracings of the contractile activity demonstrated a more robust response from SI CCS ‘tie in’ than from sham (Fig. 6h). The calculated maximum tension was significantly increased in SI CCS ‘tie in’ compared to sham, while relaxation was successfully induced at a similar level in both groups (Fig. 6i).

When evaluating epithelial function, TEER showed no significant differences between SI CCS ‘tie in’ and sham, but paracellular permeability was significantly reduced, indicating improved barrier function related to luminal content exposure (Fig. 6j). Consistent with early d5 HIO ‘tie in’ results, *I*_sc_ was reduced in SI CCS ‘tie in’ (Fig. 6j and Supplementary Fig. 3g). Furthermore, enterocyte proteins facilitating digestion, such as Peptide Transporter 1 (PEPT1, which enables di and tri-peptide absorption) and Dipeptidyl Peptidase IV (DPPIV, which degrades incretin peptides) were also observed in both groups (Supplementary Fig. 4b). Active alkaline phosphatase activity, which plays a critical role in intestinal mucosal defence, was also demonstrated in both groups (Supplementary Fig. 4c). This line of experimentation demonstrates the utility of the CCS platform for vigorous developmental modelling and manipulation. Together, these mouse and rat-derived data suggest that (1) transplanted organoids undergo substantial adaptation processes upon luminal content exposure and (2) the organoid tissues thrive and mature in physiological conditions.

## Discussion

Human gastrointestinal organoids serve as valuable models for studying development and disease. Cultured from hPSCs, these 3D systems closely resemble native tissues and have advantages to animal models. Numerous groups have been using organoids and introducing ways to improve them for basic science and translational studies^11,26,27,41–45^. The field of tissue engineering and organoid technologies is rapidly co-evolving with increased diversity in the organoids/organs that can be generated and their level of complexity^6,9,11,26,44,46–48^. Personalized medicine applications have been employed and strides towards clinical translation are underway^43,49,50^.

With this study, we have leveraged the ease of soft lithography to manufacture scaffolds that were incorporated into otherwise traditional organoid culture conditions to generate an improved organoid for transplantation. The simplicity of this experimental approach makes this methodology widely accessible and straightforward to execute. We have established a more reliable and scalable method for generating transplantable gastrointestinal tissues using this technique—some reaching 8 cm with clinically relevant epithelial surface areas^51^. Recently, centimetre-scale HIOs were developed using a rotating bioreactor, requiring an assembloid approach to combine separately generated spheroids and mesenchyme^45^. While both methods enabled elongated structures through collective behaviour, the CCS uniquely supported de novo ENS development^45^. In addition, CCS-driven maturation accelerated in vivo engraftment by 2 weeks compared to existing protocols, with enhanced efficiency—an essential factor for translating organoid technologies into clinical applications^7,11,14,26,52,53^.

Notably, CCS organoids developed a functional ENS without an assembloid approach—the addition of separately differentiated neuroglial cells, representing a paradigm shift in gastrointestinal tissue engineering. The ENS governs key functions, including motility, fluid regulation and blood flow, making its replication critical for physiological modelling. While traditionally attributed to migratory neural crest cells, there is recent data in support of the additional origins of the ENS^54–58^. While the CCS-based ENS origins remain unidentified, this model offers a unique system to investigate this and serves as a continued research direction for our groups, especially given that the CCS methodology gave rise to neuronal diversity that was previously unachieved in the assembloid HIO + ENS system^26^. Both inhibitory and excitatory neuronal subtypes were identified using snRNA-seq, protein expression analysis and electrophysiological assays. This platform could aid in understanding ENS-related diseases, such as Hirschsprung’s disease, characterized by the absence of functional ganglion cells, and may provide a neuroglial cell source for conditions involving developmental deficits^59^.

Clinical translation of organoid-based therapies requires demonstrating functional integration within physiological environments. As proof of concept, we utilized a ‘tie in’ model to confirm that CCS-derived intestinal tissues can successfully integrate with host gut architecture and function when exposed to luminal contents. This demonstrated organoids not only engraft but also adapt to host physiology, mimicking postnatal gut maturation. Barrier integrity, epithelial transport and neuromuscular activity were all maintained or enhanced, supporting their therapeutic potential for intestinal failure. In addition, the extended post-operative timeframe of the ‘tie in’ model allowed ENS components to migrate into the submucosal plexus by 22 weeks, mirroring human ENS development^60,61^. This experiment also highlighted the structural integrity and functionality of the engineered tissues in a fed intestinal state. By leveraging confinement to enhance cell–cell interactions and co-development, this methodology optimizes both in vitro and in vivo organoid maturation, providing a solid foundation for future research and therapeutic applications.

## Methods

### Manufacturing scaffolding trays

Scaffolding tray moulds were produced using a Form 2 3D printer (FormLabs) out of Surgical Guide Resin (FormLabs). Then, moulds were filled with degassed PDMS and allowed to cure before removal and use.

### Human tissue

Human tissue collection was approved by the Cincinnati Children’s Hospital Medical Center (CCHMC) Institutional Review Board (Tissue Characterization, Study No. 2014-0427; SUR iPSC and Tissue Engineering, Study No. 2017-2011). Pathologically normal human small intestine and colon samples were obtained from patients aged 14–25 years undergoing bariatric or revision/resection procedures and used as controls. Informed consent or assent was obtained from all patients and/or parent or legal guardians, as appropriate. Additional de-identified pathologically normal colon samples were obtained through the CCHMC Discover Together Biobank. All tissue use complied with institutional ethical guidelines.

### Animals

All animal procedures were approved by the CCHMC Institutional Animal Care and Use Committee (Building and Rebuilding the Human Gut, Protocol Nos. 2021-0060 and 2024-0117). Male and female adult immunodeficient Rag1^−/−^Il2rg^−/−^ rats (RRG; 3–6 months old) were used for conventional and CCS-based organoid transplantations (founders from the Transgenesis Rat ImmunoPhenomic Platform, Nantes, France; in-house breeding)^17^. Adult immunodeficient NOD-SCID IL-2Rγnull (NSG) mice (3–6 months old) were used for conventional organoid experiments (Comprehensive Mouse and Cancer Core Facility, Cincinnati, OH). Animals were housed in a barrier vivarium, with 14 h light/10 h dark cycle, ~37% humidity and temperature of 22.2 °C, and handled in accordance with the NIH Guide for the Care and Use of Laboratory Animals. One mouse was used per conventional organoid transplantation, and one rat was used per CCS organoid transplantation. Including use in all experiments and those required for breeding, 183 animals were used in the completion of this work.

### hPSC lines

The H1 human embryonic stem cell line (WA01) was obtained from WiCell. The induced pluripotent stem cell line 72_3 was generated by the CCHMC Pluripotent Stem Cell Facility and has previously been described^6^. hPSCs were cultured under feeder-free conditions using mTESR1 (StemCell Technologies) and human embryonic stem cell-qualified Matrigel (Corning). Spontaneously differentiated cells were manually removed and media were changed daily.

### Organoid generation

#### Small intestinal organoids

Aggregate and spontaneous spheroids were generated as previously described^8,14,62^. Briefly, hPSCs were differentiated to definitive endoderm using 100 ng ml^−1^ Activin A (STEMCELL Technologies), followed by hindgut induction with 100 ng ml^−1^ FGF4 (R&D Systems) and 3 µM CHIR99021 (Tocris) to generate free-floating mid-hindgut spheroids. Aggregate spheroids were additionally generated by reaggregating hindgut cells in Aggrewell 400 plates (STEMCELL Technologies). Upon collection, spheroids were embedded in growth factor-reduced (GFR) Matrigel and maintained in intestinal growth medium supplemented with 100 ng ml^−1^ EGF (R&D Systems) to generate conventional HIOs. For generation of SI CCS tissues (Bailey method), mid-hindgut spheroids were instead loaded into the confinement lanes of custom scaffolding trays at ~4,000 spheroids per confinement lane in GFR Matrigel and maintained in intestinal growth medium. After 6 days of confined culture, elongated tissues were removed from the confinement lanes and re-plated longitudinally in untreated Tissue Train culture plates (Flexcell International) using nylon tabs for anchoring, where culture continued until d14. Media were changed twice weekly for all conditions. Conventional HIOs were transplanted at d14 or d28, whereas SI CCS tissues were transplanted at d14.

#### Small intestinal organoid assembloid with neural crest cells

Organoid assembloids with an ENS (HIO + ENS) were generated as previously described^26^. Briefly, mid-hindgut spheroids were combined with neural crest cells (NCCs), which were generated in parallel to the spheroids. NCCs were differentiated as previously described for neuroglial lineages and the spheroids were generated as described above^63^. To combine the spheroids and NCCs, a ratio of 1:600 was mixed and they were centrifuged for 3 min at 300 *g* before embedding in Matrigel. The culture conditions that followed were identical to that of the conventional small intestinal organoids (HIOs) as outlined above.

#### Colonic organoids

Spheroids were generated in an identical manner as described above for small intestinal organoid generation. To distalize the structures, BMP2 (100 ng ml^−1^, R&D) was added to cultures upon plating spheroids, either conventionally or in the scaffolding trays, and changed to intestinal growth media after 72 h^7^. HCOs were utilized for surgical transplantation on d14 and d28. C CCS were utilized for transplantation on d14.

#### Stomach organoids

Spontaneous anterior foregut spheroids were generated as previously described^5,6,11^. Briefly, hPSCs were differentiated to definitive endoderm using 50 ng ml^−1^ BMP4 (R&D Systems) and 100 ng ml^−1^ Activin A (Cell Guidance Systems), followed by foregut induction with 500 ng ml^−1^ FGF4 (R&D Systems), 2 µM CHIR99021 (Tocris) and 200 ng ml^−1^ Noggin (R&D Systems). Retinoic acid (2 µM) was added on the third day of foregut induction to generate free-floating anterior foregut spheroids. Spheroids were collected on d6 and embedded in GFR Matrigel to generate conventional human antral gastric organoids (HaGOs), which were maintained in intestinal growth medium supplemented with 100 ng ml^−1^ EGF (R&D Systems), 200 ng ml^−1^ Noggin (R&D Systems) and 2 µM retinoic acid during early maturation. For generation of G CCS tissues, foregut spheroids were instead loaded into the confinement lanes of custom scaffolding trays at ~2,200 spheroids per lane in 50% GFR Matrigel and maintained under identical growth conditions. After 9 days of confined culture, elongated tissues were removed from the confinement lanes and re-plated longitudinally in untreated Tissue Train culture plates (Flexcell International) using nylon tabs for anchoring, where culture continued until d16. Media were changed twice weekly for all conditions. Conventional HaGOs were transplanted at d28, whereas G CCS tissues were transplanted at d16.

### Organoid transplantation

Conventionally generated organoids (HIOs, HIO + ENS, HCOs and HaGOs) and CCS organoids (SI CCS, C CCS and G CCS) were transplanted within the renal subcapsular space and within the mesentery as previously described^14–16^. Briefly, the kidney was accessed for kidney capsule transplantations, and a pocket was created under the kidney capsule for organoid insertion. For mesentery transplantations, the bowels were eviscerated from the thoracic cavity. A suitable bifurcating vessel was identified for the transplantation of an organoid, using a single drop of topical tissue adhesive to secure the organoid (GLUture). Due to the increased organoid size, CCS organoids were only transplanted within the mesentery. Their placement was adjacent to the host’s distal small bowel or proximal colon instead of on a bifurcating vessel structure. Both food and water were provided ad libitum before and after surgeries. Doses of carprofen (5 mg kg^−1^) and bupivacaine (1 mg kg^−1^) were administered for pain management. Animals were monitored postoperatively and additional analgesics were administered as needed.

### ‘Tie in’ surgical procedure

Ten weeks following engraftment, mice and rats underwent a secondary surgery as described previously^39^. During this ‘tie in’ procedure, the engrafted organoid (tHIO or SI CCS) was incised using a surgical scalpel blade number 11 and luminal contents removed for collection. Then, the adjacent host bowel was incised, and luminal content removed using an 18 G blunt tip fill needle and injected into the open lumen of the tHIO. Then, using a 9-0 Ethilon suture (Ethicon) the tHIO and host bowel were anastomosed in a simple interrupted fashion. Cotton swabs were used to apply an inward rolling pressure along the joined structure to expose leaks and ensure that the organoid was filled with luminal content of the host. In sham procedures, the organoid was incised, luminal contents removed before closing in a simple interrupted fashion with a 9-0 Ethilon suture. Mice were killed and tissue collected 5 d and 12 d postoperatively. Rats were killed and tissue collected 12 weeks postoperatively.

### Single-cell extractions and sequencing

Single cells were isolated from d28 HIO + ENS and d14 SI CCS in vitro structures. Organoids were carved out of their Matrigel and transferred to 1% bovine serum albumin (BSA)-coated 15 ml conical tubes. Then, the organoids were incubated in Accutase (STEMCELL Technologies) containing Y-27632 (STEMCELL Technologies) for 10 min at room temperature with intermittent pipetting to mix. After 10 min, a small portion of the mixture was visually inspected using a microscope, and an additional 2 min for digestion was allowed as needed. Then, any remaining fragments were allowed to settle and the liquid removed and added to a fresh 15 ml conical tube containing DMEMF12 with 10% FBS to stop further digestion. More Accutase containing Y-27632 was added to the remaining undigested organoid fragments, and an additional 20 min allowed for digestion. Once ~90% of the suspension was digested, DMEMF12 with 10% FBS was added to stop further digestion. Then both digestion fractions were pooled and filtered through a 40-µm cell strainer. Cells were then washed, counted and utilized for sequencing.

### scRNA-seq bioinformatics

#### scRNA-seq processing

Each sample was processed separately until the integration steps. FASTQ files were first processed and checked for quality using Cellranger (https://github.com/10XGenomics/cellranger), which aligns the reads in each sample against the human hg38 genome. The count matrix was then processed using Scanpy and a standard workflow. Doublets were estimated using the Scrublet tool^64^. Filtering consisted of the removal of all genes expressed in less than 10 cells and all cells expressing less than 200 genes, as well as cells expressing more than 5 percent of mitochondrial transcripts.

All count matrices were merged and then normalized following the shifted logarithm method. Batch-aware 3,000 highly variable genes were defined and used for downstream analysis. Data were scaled while regressing out unwanted variation from the cell cycle phase, and principal component analysis (PCA) was performed as a means of linear dimensionality reduction.

#### scRNA-seq integration and annotation

All samples were then integrated using Scanpy and the harmonypy package, using the first 50 principal components of the PCA^65^. A nearest-neighbours approach was used to compute a distance matrix and a neighbourhood graph of the cells, which was subsequently used to compute a UMAP. Clusters were computed using the Leiden algorithm^66^, and differentially expressed genes between said clusters were identified using the ‘sc.tl.rank_genes_groups()’ function of the Scanpy package. On the basis of these results and the expression of a few selected markers, clusters were then identified as one of the following populations of cells: epithelial (*EPCAM, FABP1*), mesenchymal (*COL1A2, COL3A1, MYH11, ACTG2, TAGLN*), endothelial (*CD34, PROM1, KDR, PECAM1, ICAM1, CDH5, CLDN5*) and neural (B3GAT1*, NGFR, NCAM1, PHOX2B, HAND2, TUBB2B, FOXD3, TFAP2A, ASCL1, ELAVL4, SOX10, S100B*).

#### scRNA-seq maturation scores

Using lists of known marker genes, early (*SOX10, FOXD3, PHO2B, RET, EDNRB, PAX3, NES*) and late (*ELAVL3, ELAVL4, TUBB3, DCX, MAP2, NEFL, GAP43*) neuronal scores were computed for each cell in the dataset as the mean expression of respective marker genes.

#### scRNA-seq similarity scores

To evaluate the maturity of identified neural cells, both the query and reference datasets—the Gut Cell Atlas’ subset of fetal neuronal cells—were subsetted to include only common genes^66,67^. Mean expression profiles were calculated for each annotated cell type in the reference dataset. Similarity scores were then computed for each query cell against these reference profiles using Spearman correlation. The highest similarity score and its corresponding reference cell type annotation were identified and recorded for each query cell.

### Single-nucleus extractions and sequencing

Nuclei were isolated from fresh frozen HIO, SI CCS and human jejunum tissue samples using the Minute Detergent Free Nuclei Isolation kit (Invent Biotechnologies) following manufacturer guidelines. Immediately following completion, nuclei were assessed and submitted to CCHMC’s Gene Expression Core facility to complete 10X Genomics Chromium System assays using Chromium Single Cell 3’ Library & Gel Bead Kit v3 (10X Genomics). Then, sequencing was performed by CCHMC’s DNA Sequencing and Genotyping Core on a NovaSeq 6000 System (Illumina).

### snRNA-seq bioinformatics

#### snRNA-seq processing

FASTQ files were first processed and checked for quality using Cellranger (https://github.com/10XGenomics/cellranger), which aligns the reads in each sample against the human hg38 genome. SoupX was then run on the resulting output to correct for ambient RNA^68^. The corrected counts matrix was then processed using the standard Seurat workflow^69^. Filtering removed all genes expressed in less than 3 cells and all cells with less than 200 genes. Normalization used a global scaling method to adjust and log transform the expression values for each cell, and feature selection selected the 2,000 genes with the highest cell-to-cell variation to be used in all downstream analyses. Data were also scaled to mean 0 and variance 1 while regressing out unwanted variation from mitochondrial RNA. PCA was performed as a means of linear dimensionality reduction, and cells were clustered with a *k*-nearest-neighbours approach using the first 20 principal components. A resolution of X.UMAP was also performed to visualize these clusters, a type of nonlinear dimensionality reduction. Differentially expressed genes between clusters and cell types were identified with the ‘FindAllMarkers’ function, which uses the Wilcoxon rank-sum test by default.

#### snRNA-seq integration

All samples were then integrated into a single dataset with Seurat to remove batch effects and analyse them jointly. The method involves dimensionality reduction using diagonalized canonical correlation analysis (CCA), L2 normalization of the canonical correlation vectors, and finding mutual nearest neighbours (MNNs) in the low-dimensional space between two datasets. These neighbours or ‘anchors’ were compared and used to apply a correction vector to the dataset, and this correction continued in a recursive pairwise manner between datasets until all were corrected. The first 30 canonical correlation components were used for clustering from this integrated dataset, which was then visualized with UMAP.

### Constructing a single-cell reference atlas

To annotate the cell types present in the samples, a reference atlas was first constructed using the ‘LabelHarmonization’ function in ELeFHAnt^70^. Three datasets from fetal gut development were used: E-MTAB-8901, GSE158702 and E-MTAB-10187. Intestinal cells were subsetted and combined using the CCA-based integration in the R package Seurat. We also removed certain cell types that were not informative for our data, resulting in a final dataset of 70 cell types, 62 clusters and ~115,000 cells. This dataset was divided into training and test sets, and using an ensemble learning that combines predictions from multiple classifiers, we predicted cell types for each integration cluster using ELeFHAnt. This harmonized the cell types to a final set of 41, some cell types of which were renamed, through manual curation, on the basis of marker analysis.

### Annotation of snRNA-seq

The ‘ClassifyCells_usingApproximation’ function in ELeFHAnt^70^ was used to predict cell types for the samples. The reference atlas served as the training set, samples were used as the test set, and the same ensemble-learning approach mentioned previously was applied. This assigns the most frequently predicted reference cell type within each query cluster to that cluster. As an additional annotation method, the MapQuery function in Seurat was used. Following a similar method as seen with integration, similar pairs of cells or ‘anchors’ were found between reference and query by projecting the reference PCA onto the query rather than using CCA. Using these anchors, a weighted vote classifier found the highest confidence cell type in the reference to assign to each query cell. Finally, these predictions were visualized by projecting the query onto the reference UMAP.

### Bulk RNA isolation and sequencing

According to manufacturer guidelines, RNA was extracted from in vitro organoids using an RNeasy Plus Micro kit (Qiagen). Then, samples were quantified and submitted to CCHMC’s DNA Sequencing and Genotyping Core for next generation sequencing. All samples were assayed to have RNA integrity numbers greater than 8. After quality control, a cDNA library was created and sequenced using an Illumina HiSeq 2000 system with 20 million paired-end reads per sample.

#### Bulk RNA-seq processing

FASTQ files were processed with CSBB (https://github.com/csbbcompbio/CSBB-v3.0), which uses RSEM^71^ for mapping and quantification. Paired-end reads were aligned against the human reference genome hg38 using bowtie2. All samples were checked for quality using FastQC metrics (https://www.bioinformatics.babraham.ac.uk/projects/fastqc/).

#### Bulk RNA-seq analysis

The count matrices generated were first filtered to remove long non-coding RNAs. DESeq2 (ref. ^72^) was then used to output differentially expressed genes by comparing genes between conditions of interest. For PCA, the normalized counts matrix (in transcripts per million) was log transformed and principal components were calculated. Figures for the PCA loadings were produced using the R package pcaExplorer^73^. Heat maps of differentially expressed genes were generated using log-transformed counts with the R package pheatmap (https://cran.r-project.org/web/packages/pheatmap/index.html).

### Tissue processing and immunostaining

Tissue samples were handled and processed as previously described^12^. Briefly, tissues were fixed overnight in 4% paraformaldehyde at 4 °C, processed using a HistoCore Pearl tissue processor (Leica) on a 12-h cycle and embedded in paraffin. Sections were deparaffinized, rehydrated, and either stained with haematoxylin and eosin (H&E) or subjected to heat-induced epitope retrieval followed by immunostaining. Blocking was performed using 10% normal serum, and primary antibodies were incubated overnight at 4 °C in 1% BSA in phosphate-buffered saline (PBS). Antibodies and dilutions are listed in Supplementary Table 1. Signal amplification was performed using the Vectastain ABC system with diaminobenzidine detection (Vector Laboratories). Active alkaline phosphatase activity was detected using the Vector Red kit without antibody incubation or blocking. Lillie–Mayer’s haematoxylin (Agilent Technologies) was used as a counterstain.

### Image acquisition

Surgical images were acquired using an M80 microscope with an MC 170HD camera (Leica Microsystems). Gross images of collected tissues were obtained using a V60 ThinQ smartphone (LG Electronics). Tissue collections were performed under an M165 FC microscope equipped with a DCF7000 T camera (Leica Microsystems). Histological slides were imaged using an Eclipse Ti microscope, and analyses were conducted using Nikon Elements Imaging Software.

### Organoid size and morphometric analysis

Engrafted organoids were photographed with a ruler and quantified using ImageJ (National Institutes of Health) with a conversion factor of 1 mm. Morphometric analysis was performed on H&E-stained tissue sections. Crypt depth, crypt width, villus height, villus width and mucosal thickness were measured for a minimum of 20 well-oriented crypt–villus units per sample and averaged using Nikon NIS imaging software. Measurements were collected across the entire tissue section without selection of specific regions, and each data point represents the average of a biological replicate. Neuronal bundle size was quantified by measuring cross-sectional areas across entire tissue sections using Nikon NIS software. For goblet cell analysis, three ×20 fields of view of Alcian blue-stained sections were acquired per biological replicate. Goblet cells were counted and normalized to epithelial length using the Annotations and Measurements tools in Nikon NIS software.

### Ex vivo neuromuscular function and muscle tone

Organ bath chamber assays were performed as previously described^11,12,26^. Muscularis from freshly collected organoid samples was dissected into strips (~2 × 6 mm) without chelation and mounted in an organ bath system (Radnoti) connected to force transducers (ADInstruments). Contractile activity was recorded continuously using LabChart software (ADInstruments). Following equilibration, cumulative concentration–response curves were generated using carbamyl-β-methylcholine chloride (bethanechol, Sigma-Aldrich) at concentrations ranging from 1 nM to 10 mM at 2-min intervals, followed by administration of 10 µM scopolamine (Tocris Bioscience).

After re-equilibration, tissues were subjected to EFS using a control pulse (8,000 mV for 400 µs, −1,000 mV for 400 µs, followed by a 400-µs rest, repeated for 10 s). L-NAME (50 µM, Sigma-Aldrich) was applied 10 min before EFS to assess nitric oxide synthase inhibition, followed by atropine (1 µM, Sigma-Aldrich) without washout to assess combined nitrergic and cholinergic inhibition. After washout and re-equilibration, a control EFS pulse was repeated. TTX (4 µM, Tocris Bioscience) was applied 5 min before a final EFS stimulation. Responses were quantified as the area under the curve (AUC) for 60 s before and after stimulation. Cholinergic, nitrergic and non-cholinergic/non-nitrergic components were calculated by sequential subtraction of responses following L-NAME and atropine treatment relative to total responses. All data were normalized to muscle strip mass.

### Ex vivo epithelial characteristics and permeability

The epithelia of freshly collected sham and ‘tie in’ grafts were carefully dissected through a technique similar to seromuscular stripping as previously reported^12,74,75^. Tissue was incised and dissected in ice-cold Krebs buffer (NaCl 117 mM, KCl 4.7 mM, MgCl_2_ 1.2 mM, NaH_2_PO_4_ 1.2 mM, NaHCO_3_ 25 mM, CaCl_2_ 2.5 mM, glucose 11 mM). Full-thickness segments were pinned in Sylgard-coated dishes (Electron Microscopy Sciences), and the seromusculature was micro-dissected from the epithelium using Dumont #5 and #7 forceps and Vannas scissors (Fine Science Tools). Tissue integrity was assessed by stereomicroscopy, and damaged regions were excluded. Residual subepithelial mucosa remained, as previously reported^12^. The central epithelial region was mounted in an Ussing chamber (Physiologic Instruments), exposing 0.008 cm^2^ of tissue to 3 ml oxygenated Krebs buffer at 37 °C. Transepithelial potential difference was measured using paired electrodes connected via agar–KCl salt bridges and interfaced with a VVC MC8 voltage clamp amplifier (Physiologic Instruments). Baseline offsets were zeroed before mounting, followed by a 30-min equilibration period. Tissues were voltage clamped at 0 mV, and *I*_sc_ was recorded continuously. For permeability assays, FITC–dextran (2.2 mg ml^−1^) was added to the apical compartment, and basolateral samples were collected every 30 min for 3 h with buffer replacement. FITC–dextran concentrations were quantified using a Synergy 2 plate reader (BioTek).

### Data representation, statistics and reproducibility

For bar graphs, data are represented as mean ± s.d., with all individual data points represented where possible. For violin plots, a dashed line indicates the mean and dotted lines the interquartile ranges, representing all individual data points. Statistical significance cut-off was *P* < 0.05 and confidence was 95%.

### Reporting summary

Further information on research design is available in the Nature Portfolio Reporting Summary linked to this article.

## Supplementary information

Supplementary Information Supplementary Figs. 1–4 and Table 1.

Reporting Summary

Peer Review File

## Source data

Source Data Fig. 1 Statistical source data.

Source Data Fig. 3 Statistical source data.

Source Data Fig. 5 Statistical source data.

Source Data Fig. 6 Statistical source data.

Source Data Extended Data Fig. 1 Statistical source data.

Source Data Extended Data Fig. 3 Statistical source data.

## Data Availability

All data supporting the findings of this study are available within the paper and its Supplementary Information. Single-cell and bulk sequencing data have been made publicly available on the European Nucleotide Archive (ENA) under project accession PRJEB84049. Single-nucleus sequencing data have been made publicly available on the National Center for Biotechnology Information (NCBI) GEO series under project accession GSE233439. Three datasets from fetal gut development were used to create a reference: E-MTAB-8901, GSE158702 and E-MTAB-10187 from the Wellcome Sanger Institute Gut Cell Survey. In addition, study SCP1038 from the Broad Institute’s Single-Cell Portal was utilized. Source data are provided with this paper.
