# Supplementary material for: Large-scale and innervated functional human gut tissues for transplantation via transient spheroid confinement
Source: Nat Biomed Eng. 2026 May 22;10(10):2077–93. doi: 10.1038/s41551-026-01688-6 (PMC13672046; doi:10.1038/s41551-026-01688-6)
Supplement: Supplementary file 1 [file 41551_2026_1688_MOESM1_ESM.pdf]

nature biomedical engineering

Supplementary information

https://doi.org/10.1038/s41551-026-01688-6

Large-scale and innervated functional human gut tissues for transplantation via transient spheroid confinement

In the format provided by the authors and unedited

Supplemental Information

Table of Contents

Supplemental Figure 1……………………………………………………………………2
Scaffolding tray design and in vitro SI CCS growth.

Supplemental Figure 2……………………………………………………………………3
Neurogenesis is enhanced in SI CCS prior to transplantation.

Supplemental Figure 3……………………………………………………………………5
Modeling the short-term fed intestinal state can be done with HIOs in mice but is associated with high mortality.

Supplemental Figure 4……………………………………………………………….......7
SI CCS have reduced goblet cell frequency and preserved enterocyte marker expression in the long-term fed intestinal state.

Supplemental Table 1…………………………..…………………………………………8
List of antibodies used for immunostaining.

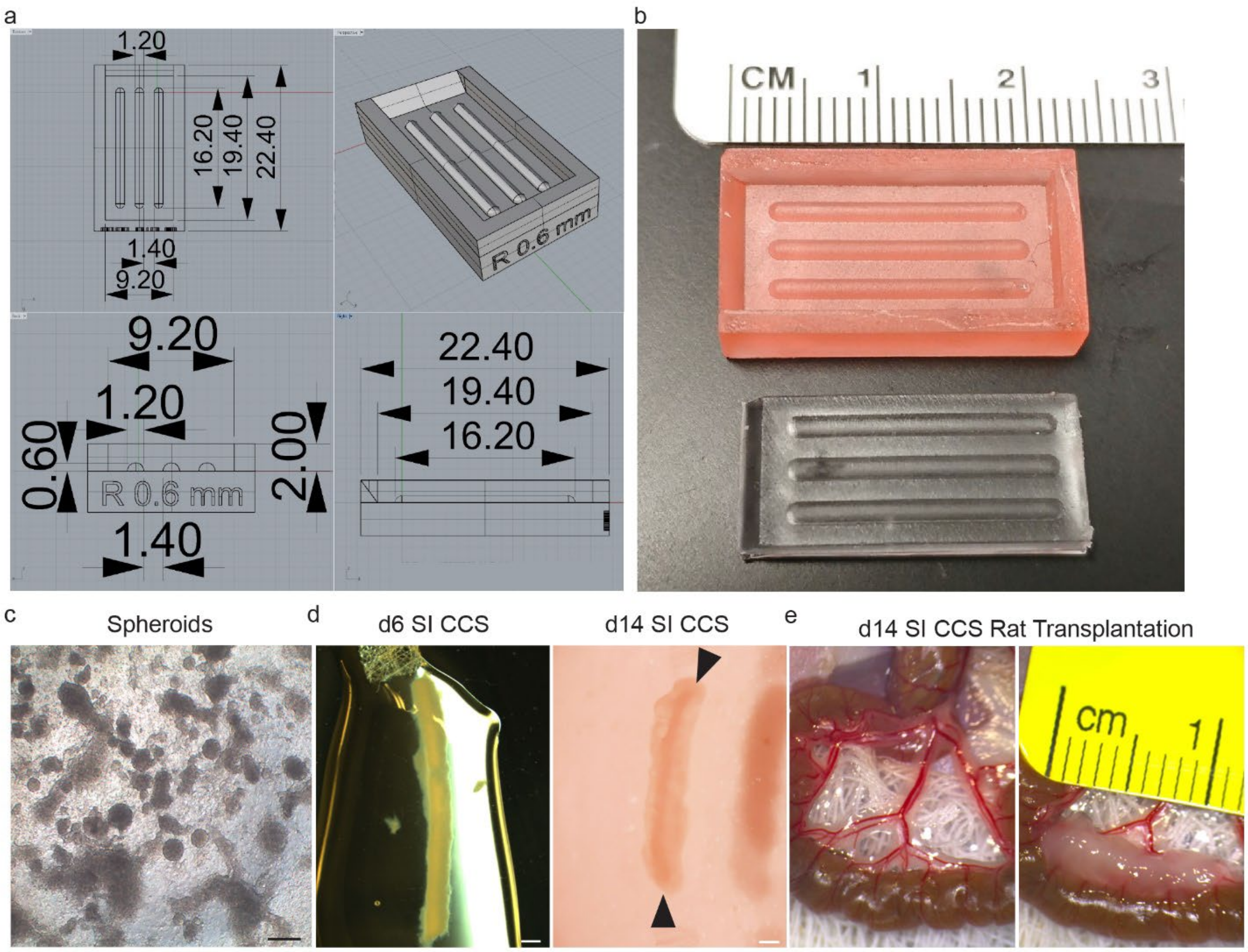

Supplemental Figure 1. Scaffolding tray design and in vitro SI CCS growth.
a, Schematic of scaffolding tray mold. **b**, Photograph of 3D printed scaffolding tray mold and PDMS scaffolding tray. **c**, Images of spheroids generated by spontaneous morphogenesis. Scale bars = 100 µm. **d**, Representative image of freshly replated d6 SI CCS after removal from scaffolding tray (left) and d14 SI CCS (right). Arrows indicate ends of dark longitudinal luminal area. Scale bars = 1 mm. **e**, Photographs of d14 SI CCS transplantation. **f**, Photograph demonstrating the central lumen after 10 weeks of engraftment.

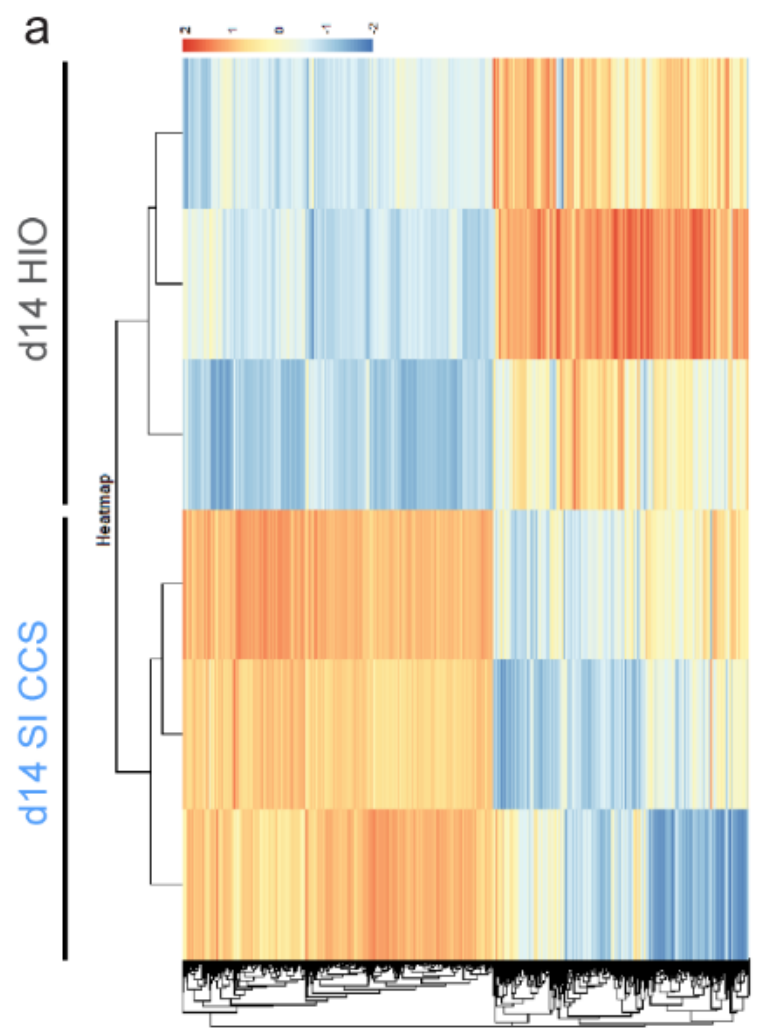

b

Ontology	Biological Process	P Value
GO:0022008	neurogenesis	2.59E-44
GO:0048699	generation of neurons	7.02E-43
GO:0030182	neuron differentiation	7.89E-42
GO:0007471	central nervous system development	2.07E-33
GO:0007049	cell cycle	3.14E-33

Ontology	Cellular Component	P Value
GO:0030424	axon	1.44E-25
GO:0045202	synapse	7.26E-24
GO:0043005	neuron projection	3.15E-23
GO:0097060	synaptic membrane	8.56E-22
GO:0098794	post synapse	9.10E-22

c

Events Captured in Single Cell Transcriptomics

Organoid	Cell Type	Number
HIO+ENS	endothelial	3
	epithelial	1,666
	mesenchymal	4,152
	neural	3,193
	total	9,014
SI CCS	endothelial	69
	epithelial	2,612
	mesenchymal	33,869
	neural	1,352
	total	37,902

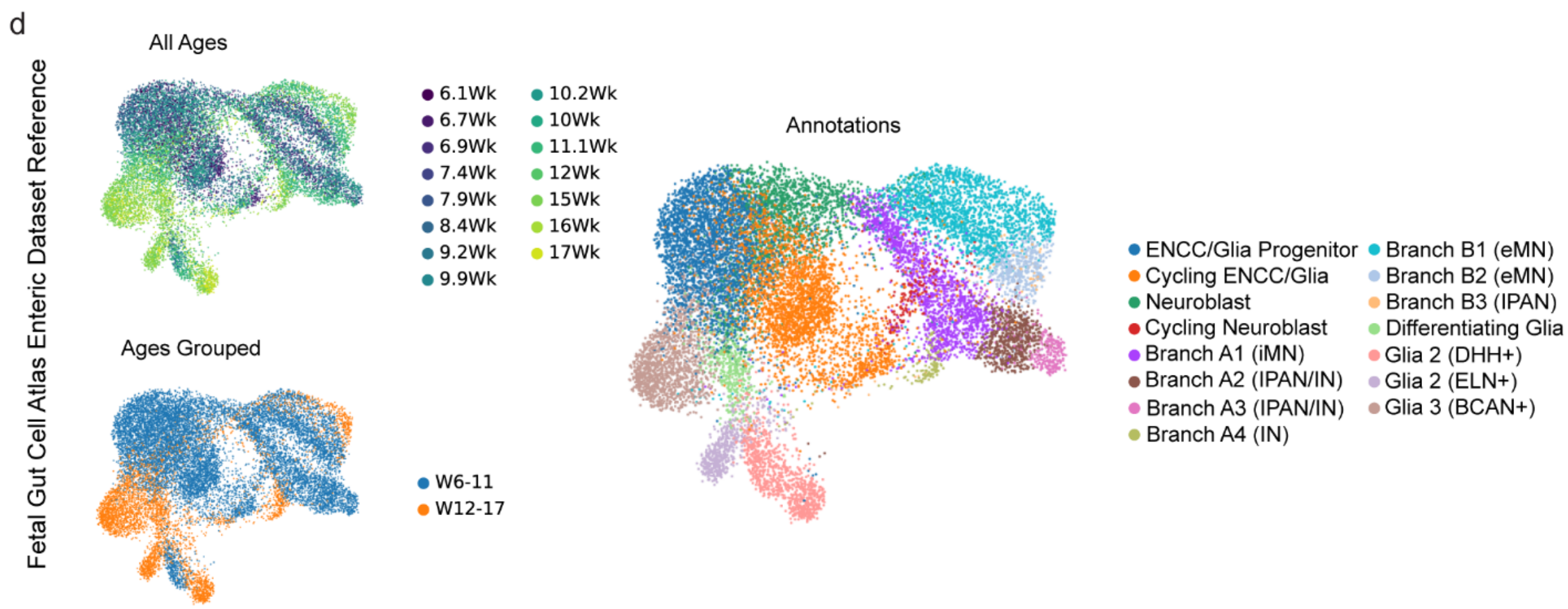

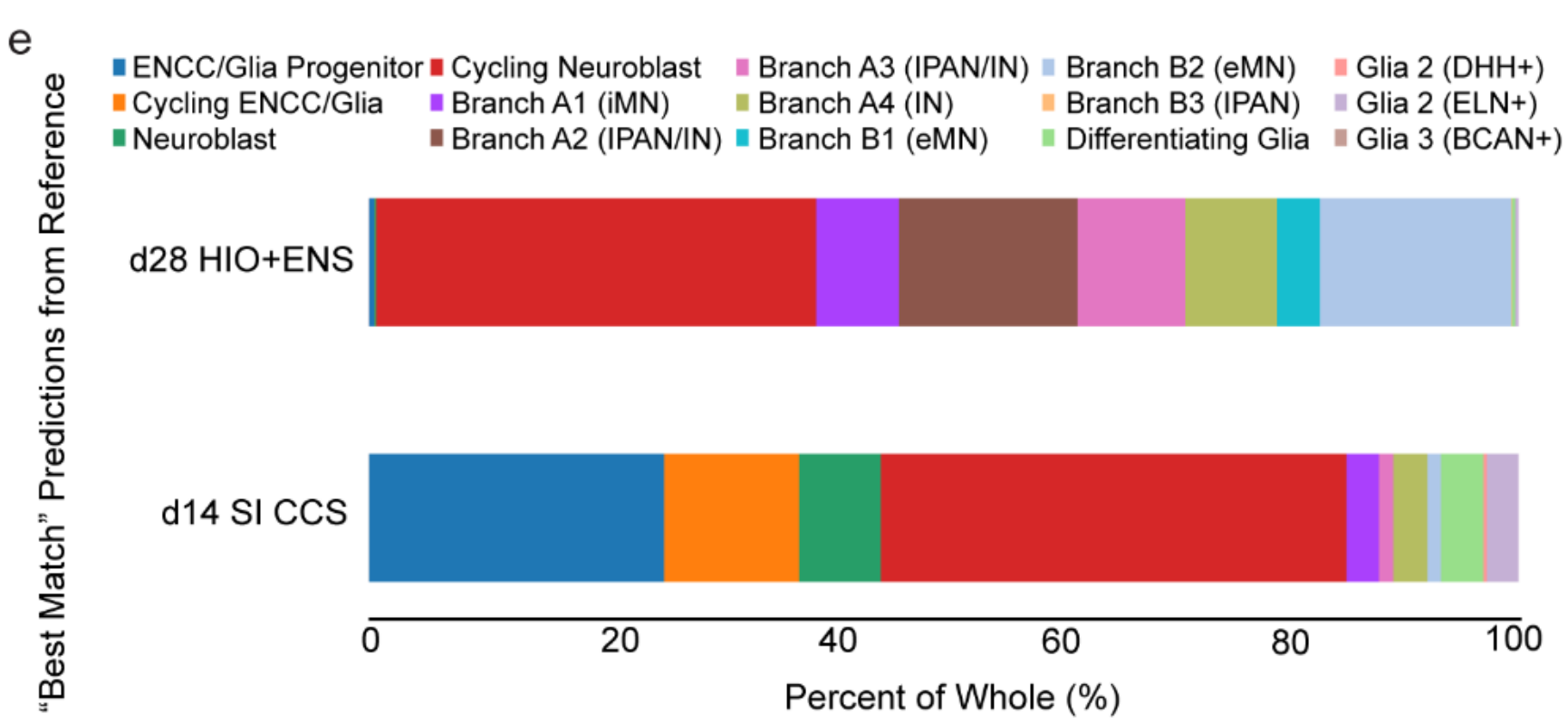

	d28 HIO+ENS	d14 SI CCS
ENCC/Glia Progenitor	14	347
Cycling ENCC/Glia	1	159
Neuroblast	6	96
Cycling Neuroblast	1221	548
Branch A1 (iMN)	231	38
Branch A2 (IPAN/IN)	496	0
Branch A3 (IPAN/IN)	299	17
Branch A4 (IN)	254	40
Branch B1 (eMN)	119	0
Branch B2 (eMN)	532	16
Branch B3 (IPAN)	2	0
Differentiating glia	9	50
Glia 1 (DHH+)	0	4
Glia 2 (ELN+)	9	37
Glia 3 (BCAN+)	0	0

Supplementary Figure 2. Neurogenesis is enhanced in SI CCS prior to transplantation.
a, Heatmap of bulkRNAseq data in d14 HIO and d14 SI CCS. **b**, Un-curated list of enriched gene ontologies in d14 SI CCS compared to d14 HIO. **c**, Table of cell type designations for scRNAseq analysis of d28 HIO+ENS and d14 SI CCS and their corresponding event (cell) counts. **d**, Fetal Gut Cell Atlas enteric reference curation into two time frames (early, w6-11, and late, w12-17)

and associated cell type annotations. **e**, Bar graph and table of “Best Match” neural cell type predictions from the curated Gut Cell Atlas reference for d28 HIO+ENS and d14 SI CCS.

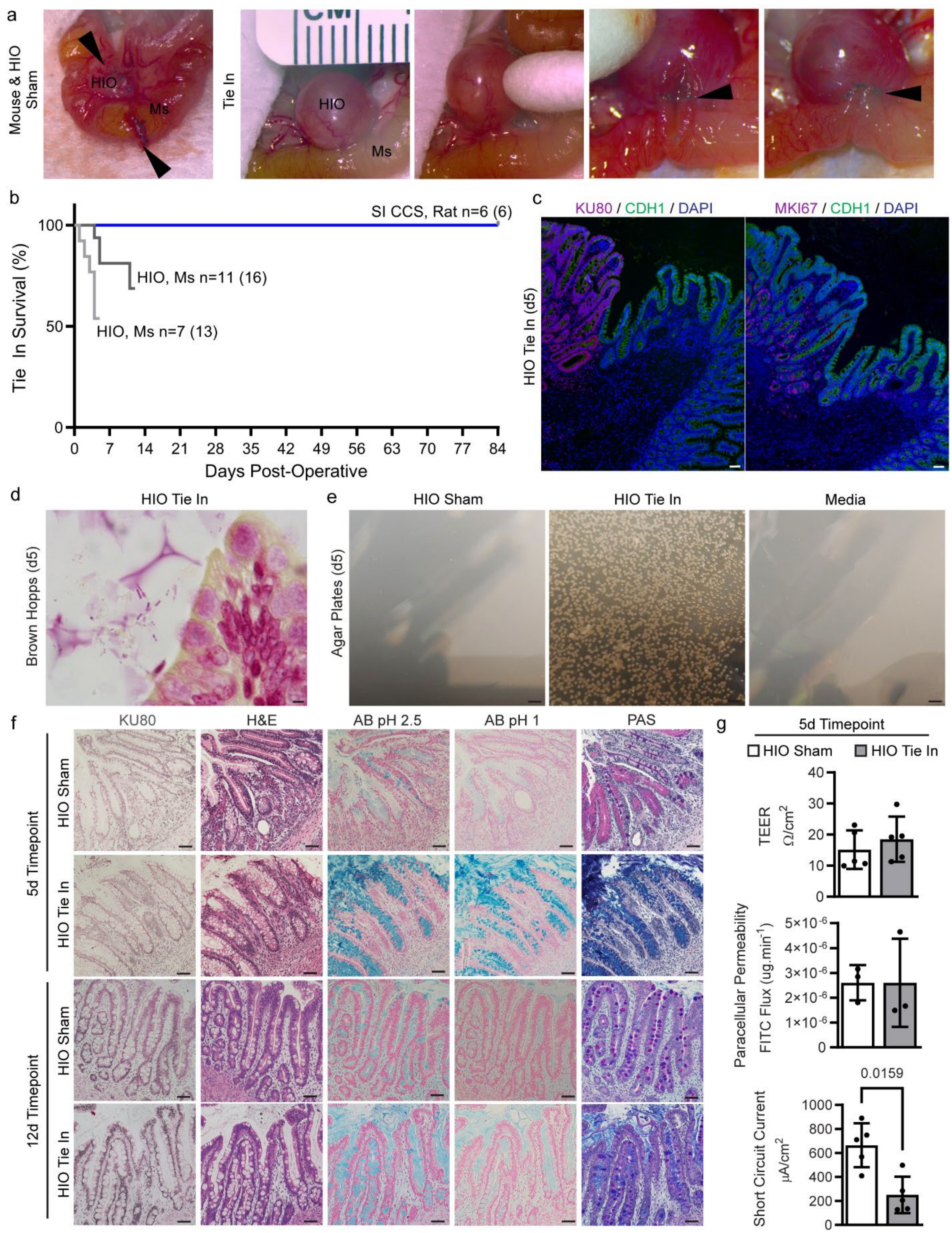

Supplementary Figure 3. Modeling the short-term fed intestinal state can be done with HIOs in mice, but is associated with high mortality.

a, Surgical images of HIO Sham and Tie In procedures. Sites of anastomosis are marked with black arrowheads. HIO and mouse tissues are denoted with text. **b**, Kaplan-Meier curve of survival associated with 5 day HIO Tie Ins, 12 day HIO Tie Ins and 12 week SI CCS Tie Ins. c, Representative immunofluorescence staining for human cells (KU80, magenta) and epithelium (CDH1, green) demonstrating continuity between organoid and host (left). Serial section stained for proliferation (MKI67, magenta) and epithelium (green) demonstrating proliferative zonation within the intestinal epithelium. Scale bars = 50 μm. **d**, Representative Brown-Hopps staining for bacteria in HIO Tie In demonstrating bacteria near the epithelium. Scale bars = 5 μm. **e**, Photographs of agar plates after 72 hours of culture using luminal contents from HIO Sham, HIO Tie In, and media alone as a negative control. Colony outgrowth was only visible in HIO Tie In samples. Scale bars = 5 mm. **f**, Representative staining panels of mucin profiles across HIO Sham and Tie In at the 5d and 12d timepoints. A human marker (KU80, gray) was used to validate tissue source in serial sectioned panels of histological stains (left to right: H&E, alcian blue pH 2.5, alcian blue pH 1.0, and Periodic acid-Schiff). Scale bars = 50 μm. **g**, Bar graphs (mean±SD) of functional epithelial characteristics in HIO Sham and HIO Tie In at d5 as measured in an Ussing Chamber assay. From top to bottom: transepithelial resistance, paracellular permeability and short circuit current. n ≥ 3 per group for staining and n = 5 per group for electrophysiology measurements.

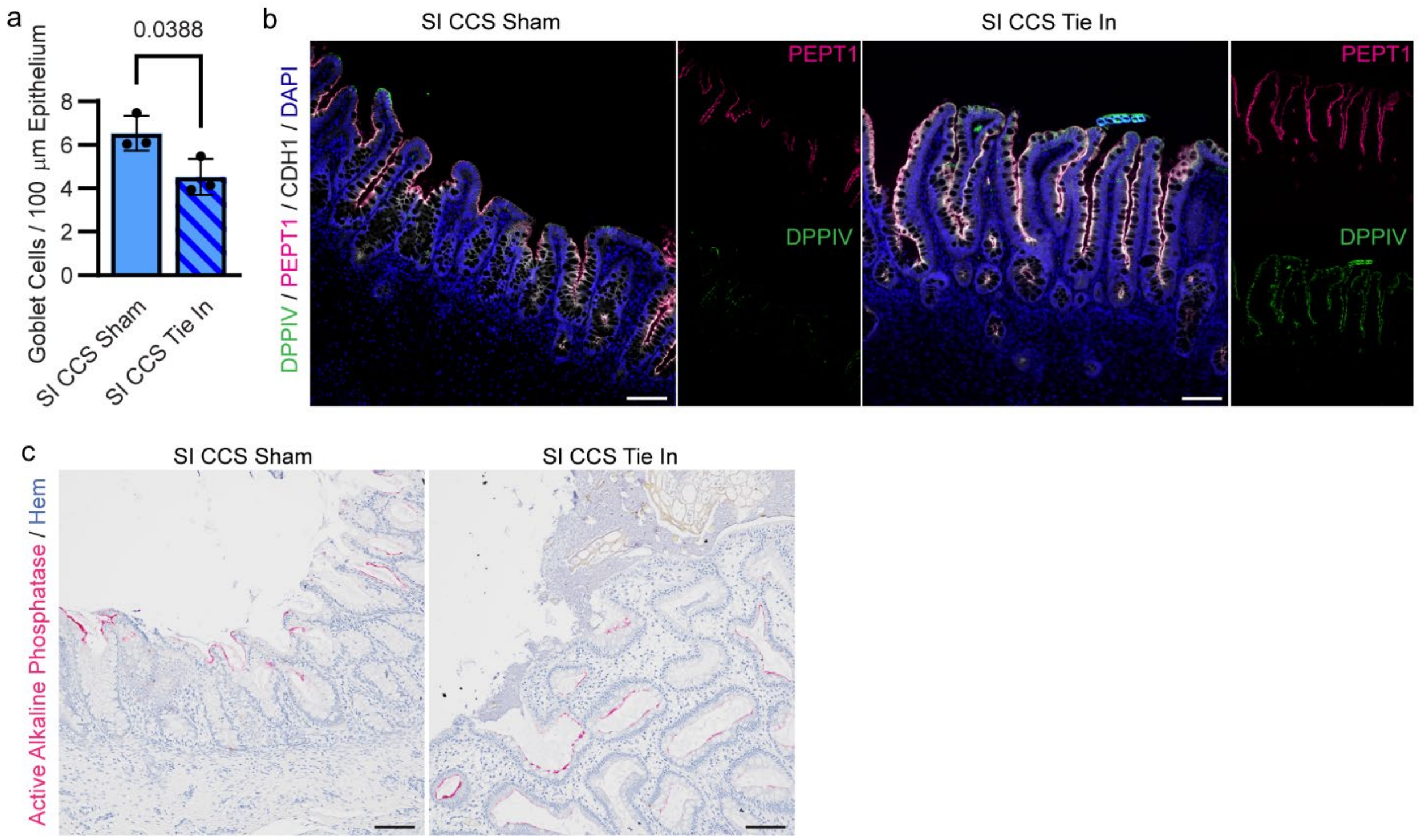

Supplementary Figure 4. SI CCS have reduced goblet cell frequency and preserved enterocyte marker expression in the long-term fed intestinal state.

a, Bar graph (mean±SD) of goblet cell quantification in SI CCS Sham and Tie In. Two-tailed Student T test performed. **b**, Representative immunofluorescence staining for a brush border enzyme (DPPIV, green), brush border transporter (PEPT1, pink) and epithelium (CDH1, white) in SI CCS Sham and Tie In. Single channels of the brush boarder enzyme and transporter staining are also presented for clarity. **c**, Representative images of active alkaline phosphatase activity in SI CCS Sham and Tie in. n = 4 per group for staining. Scale bars = 100 μm

	Antigen	Dilution	Host	Company: Catalog Number
**Primary**	ATP4B	1:500	mouse	Invitrogen: MA3-923
	CA1	1:800	rat	R&D: MAB2180
	CD31	1:200	rabbit	Abcam: ab28364
	CDH1	1:500	mouse	BD: 610182
	CHAT	1:200	rabbit	Millipore: AB-143
	CHGA	1:500	mouse	DSHB: CPTC-CHGA-1
	CLDN18	1:800	rabbit	Atlas: HPA018446
	DEFA5	1:500	mouse	Abcam: ab90802
	DPPIV	1:800	goat	R&D: AF1180
	F3	1:200	rabbit	Atlas: HPA049292
	GHRL	1:500	goat	Novus: NB600-813
	HuC / HuD	1:200	mouse	Invitrogen: A21271
	KIT	1:300	rabbit	Abcam: ab32363
	MKI67	1:500	rabbit	Thermo: RM-9106-S0
	MUC5B	1:900	rabbit	Atlas: HPA008246
	MUC6	1:1000	mouse	Novus: NBP2-47799
	NES	1:400	mouse	Abcam: ab22035
	NF200	1:2000	chicken	Antibodies-Online: ABIN953663
	NOS1	1:200	rabbit	Abcam: ab76067
	PEPT1	1:200	rabbit	Santa Cruz: sc-20653
	S100B	1:300	rabbit	Atlas: AMAb91038
	SATB2	1:200	rabbit	Millipore Sigma: 384R-14
	SI	1:1000	rabbit	Thermo: HPA011897
	SOX2	1:500	rabbit	GeneTex: GTX101507
	SYN1	1:300	rabbit	Abcam: ab8
	TUBB3	1:350	chicken	Abcam: ab41489
	UCHL1 / PGP9.5	1:750	rabbit	Dako: Z511601
	XRCC5 / KU80	1:350	rabbit	Cell Signaling: 2180
**Secondary**	anti-chicken AF555	1:1000	donkey	Thermo: A21449
	anti-goat AF647	1:1000	donkey	Thermo: A21447
	anti-mouse AF555	1:1000	donkey	Thermo: A21137
	anti-rabbit AF488	1:1000	donkey	Thermo: A21206
	anti-rat AF488	1:1000	donkey	Thermo: A21208

Supplemental Table 1. List of antibodies used for immunostaining.
