# Supplementary material for: Large-scale and innervated functional human gut tissues for transplantation via transient spheroid confinement
Source: Nat Biomed Eng. 2026 May 22;10(10):2077–93. doi: 10.1038/s41551-026-01688-6 (PMC13672046; doi:10.1038/s41551-026-01688-6)
Supplement: Supplementary file 2 [file 41551_2026_1688_MOESM2_ESM.pdf]

nature portfolio

Corresponding author(s): Michael A Helmrath
Maxime M Mahe

Last updated by author(s): Jan 14, 2026

# Reporting Summary

Nature Portfolio wishes to improve the reproducibility of the work that we publish. This form provides structure for consistency and transparency in reporting. For further information on Nature Portfolio policies, see our Editorial Policies and the Editorial Policy Checklist.

## Statistics

For all statistical analyses, confirm that the following items are present in the figure legend, table legend, main text, or Methods section.

| n/a | Confirmed | |
|---|---|---|
| ☐ | ☒ | The exact sample size (*n*) for each experimental group/condition, given as a discrete number and unit of measurement |
| ☐ | ☒ | A statement on whether measurements were taken from distinct samples or whether the same sample was measured repeatedly |
| ☐ | ☒ | The statistical test(s) used AND whether they are one- or two-sided<br>*Only common tests should be described solely by name; describe more complex techniques in the Methods section.* |
| ☐ | ☒ | A description of all covariates tested |
| ☐ | ☒ | A description of any assumptions or corrections, such as tests of normality and adjustment for multiple comparisons |
| ☐ | ☒ | A full description of the statistical parameters including central tendency (e.g. means) or other basic estimates (e.g. regression coefficient) AND variation (e.g. standard deviation) or associated estimates of uncertainty (e.g. confidence intervals) |
| ☐ | ☒ | For null hypothesis testing, the test statistic (e.g. *F*, *t*, *r*) with confidence intervals, effect sizes, degrees of freedom and *P* value noted<br>*Give P values as exact values whenever suitable.* |
| ☒ | ☐ | For Bayesian analysis, information on the choice of priors and Markov chain Monte Carlo settings |
| ☒ | ☐ | For hierarchical and complex designs, identification of the appropriate level for tests and full reporting of outcomes |
| ☒ | ☐ | Estimates of effect sizes (e.g. Cohen's *d*, Pearson's *r*), indicating how they were calculated |

*Our web collection on statistics for biologists contains articles on many of the points above.*

## Software and code

Policy information about availability of computer code

| | |
|---|---|
| Data collection | Nikon NIS Elements Advanced Research 6.02.01, Image J 1.53a, LabChart Pro 8.1.30 software programs were used in the collection of our data |
| Data analysis | Graphpad Prism 10.4.1, Nikon NIS Elements Advanced Research 6.02.01, Image J 1.53a, LabChart Pro 8.1.30, Microsoft Excel 365 Enterprise 2512, R 4.4.0 and RStudio Mariposa Orchid software programs and associated packages were used in the analysis of our data. R and RStudio packages used are detailed within the methods section. |

For manuscripts utilizing custom algorithms or software that are central to the research but not yet described in published literature, software must be made available to editors and reviewers. We strongly encourage code deposition in a community repository (e.g. GitHub). See the Nature Portfolio guidelines for submitting code & software for further information.

## Data

Policy information about availability of data

All manuscripts must include a data availability statement. This statement should provide the following information, where applicable:

- Accession codes, unique identifiers, or web links for publicly available datasets
- A description of any restrictions on data availability
- For clinical datasets or third party data, please ensure that the statement adheres to our policy

The authors declare that all data supporting the findings of this study are available within the paper and its supplementary information. Single cell and bulk bequencing data have been made publicly available on the European Nucleotide Archive (ENA) under project accession PRJEB84049. Single nucleus sequencing data

have been made publicly available on the National Center for Biotechnology Information (NCBI) GEO series under project accession GSE233439. Three datasets from fetal gut development were used to create a reference: E-MTAB-8901, GSE158702, and E-MTAB-10187 from the Wellcome Sanger Institute Gut Cell Survey. Additionally, study SCP1038 from the Broad Institute's Single Cell Portal was utilized.

## Research involving human participants, their data, or biological material

Policy information about studies with human participants or human data. See also policy information about sex, gender (identity/presentation), and sexual orientation and race, ethnicity and racism.

| | |
|---|---|
| Reporting on sex and gender | Samples from both sexes were collected and no preference was given toward this characteristic when recruiting individuals. Sex and gender-based analysis was not preformed, due to small sample size for assays (e.g. single nuclear RNA sequencing was done on one human intestinal tissue sample). Gender identity was not collected for this study, nor was consent for sharing individual-level sex and gender information. |
| Reporting on race, ethnicity, or other socially relevant groupings | No selection criteria related to race, ethnicity or other social groupings was used during this study. A mix of samples were collected from Caucasian and Hispanic non-Caucasian identifying individuals, which was based on the patient population receiving bariatric surgical procedures that consented to participate in the study. |
| Population characteristics | The patient population in this study was used as a benchmark control for gastrointestinal tissue types. Patients with obesity were undergoing bariatric surgical procedures, but were otherwise generally healthy individuals. Patients were of the ages 7 to 16 years old at the time of sample collection. |
| Recruitment | Clinical research coordinators recruited individuals undergoing surgery (without known chromosomal or genetic conditions) and obtained informed consent/HIPAA authorization prior to the procedure to collect otherwise discarded tissue; care and surgical plans were unchanged and the risk assumed minimal. Specimens were given identification numbers and de-identified for laboratory use. Human donor tissues were included only to anchor measurements in a human context; as our inferences relyed on predefined analyses of the engineered organoid constructs, any selection bias in this small reference set is unlikely to alter the conclusions. |
| Ethics oversight | Cincinnati Children's Hospital Medical Center's Internal Review Board approved our human subjects research. |

Note that full information on the approval of the study protocol must also be provided in the manuscript.

# Field-specific reporting

Please select the one below that is the best fit for your research. If you are not sure, read the appropriate sections before making your selection.

☒ Life sciences ☐ Behavioural & social sciences ☐ Ecological, evolutionary & environmental sciences

For a reference copy of the document with all sections, see nature.com/documents/nr-reporting-summary-flat.pdf

# Life sciences study design

All studies must disclose on these points even when the disclosure is negative.

| | |
|---|---|
| Sample size | Given the novelty of this technique a power calculation could not be performed, however based on previous results from our group we estimated the initial sample size as five organoids per per group because we have achieved statistical significance with this sample size previously. From there, our transplantation numbers were increased as needed in order to have sufficient tissue for each assay. In the case of human tissue we were limited by availability. |
| Data exclusions | No data was excluded.<br>Bioinformatic approaches were utilized to minimize mitochondrial RNA contamination, but the raw datasets are available. |
| Replication | Replication attempts were successful across a minimum of three separate differentiations for all organoid types. |
| Randomization | hPSC derived spheroids were randomly made into either conventional organoids or seeded into the scaffolding trays to generate groove-based confined culture system organoids. The starting material was identical and no criteria contributed to the manner a spheroid was matured. |
| Blinding | Two individuals independently collected data and were blinded to sample groups/type where possible, e.g. morphomorphic image based quantification and immunostaining processes. At the time of harvest it was impossible to be blinded to group because of drastic phenotypic differences, including during the microdissections for physiologic assays. A number based identification system was used for all samples (paraffin blocks, slide labels, frozen tissue samples, and live tracings) to enhance the amount of blinding throughout all data acquisition and analysis. |

# Reporting for specific materials, systems and methods

We require information from authors about some types of materials, experimental systems and methods used in many studies. Here, indicate whether each material, system or method listed is relevant to your study. If you are not sure if a list item applies to your research, read the appropriate section before selecting a response.

## Materials & experimental systems

| n/a | Involved in the study |
|---|---|
| ☐ | ☒ Antibodies |
| ☐ | ☒ Eukaryotic cell lines |
| ☒ | ☐ Palaeontology and archaeology |
| ☐ | ☒ Animals and other organisms |
| ☒ | ☐ Clinical data |
| ☒ | ☐ Dual use research of concern |
| ☒ | ☐ Plants |

## Methods

| n/a | Involved in the study |
|---|---|
| ☒ | ☐ ChIP-seq |
| ☒ | ☐ Flow cytometry |
| ☒ | ☐ MRI-based neuroimaging |

# Antibodies

Antibodies used

ATP4B Invitrogen: MA3-923, 1:500
CA1 R&D: MAB2180, 1:800
CD31 Abcam: ab28364, 1:200
CDH1 BD: 610182, 1:500
CHAT Millipore: AB-143, 1:200
CHGA DSHB: CPTC-CHGA-1, 1:500
CLDN18 Atlas: HPA018446, 1:800
DEFA5 Abcam: ab90802, 1:500
DPPIV R&D: AF1180, 1:800
F3 Atlas: HPA049292, 1:200
GHRL Novus: NB600-813, 1:500
HuC / HuD Invitrogen: A21271, 1:200
KIT Abcam: ab32363, 1:300
MKI67 Thermo: RM-9106-S0, 1:500
MUC5B Atlas: HPA008246, 1:900
MUC6 Novus: NBP2-47799, 1:1000
NES Abcam: ab22035, 1:400
NF200 Antibodies-Online: ABIN953663, 1:2000
NOS1 Abcam: ab76067, 1:200
PEPT1 Santa Cruz: sc-20653, 1:200
S100B Atlas: AMAb91038, 1:300
SATB2 Millipore Sigma: 384R-14, 1:200
SI Thermo: HPA011897, 1:1000
SOX2 GeneTex: GTX101507, 1:500
SYN1 Abcam: ab8, 1:300
TUBB3 Abcam: ab41489, 1:350
UCHL1 / PGP9.5 Dako: Z511601, 1:750
XRCC5 / KU80 Cell Signaling: 2180, 1:350
anti-chicken AF555 Thermo: A21449, 1:1000
anti-goat AF647 Thermo: A21447, 1:1000
anti-mouse AF555 Thermo: A21137, 1:1000
anti-rabbit AF488 Thermo: A21206, 1:1000
anti-rat AF488 Thermo: A21208, 1:1000

Validation

All primary and secondary antibodies were chosen based off of inclusion in previous publication, followed by in lab validation on human gastric, intestinal, and colonic tissue as necessary prior to use on experimental samples. Antibodies, their dilutions, host species and catalogue numbers are also listed in Supplementary Table 1 for reader convenience.

# Eukaryotic cell lines

Policy information about cell lines and Sex and Gender in Research

Cell line source(s)

The H1 line (male) of human embryonic stem cells was used in this study and obtained from WiCell Research Institute, Inc (reference WA01). The induced pluripotent stem cell line 72_3 (male) was generated by the CCHMC Pluripotent Stem Cell Facility and has previously been described.

Authentication

Pluripotentcy was assessed by pluripotentcy marker expression (OCT4, Nanog) and morphology. Karyotyping and STR analysis was also carried out for line 72_3.

Mycoplasma contamination

Lab personnel regularly test for Mycoplasma contamination in house using the MycoAlert kit from Lonza. Only cultures free of contamination were used to generate the tissue constructs in this study.

Commonly misidentified lines
(See ICLAC register)

No commonly misidentified lines were used.

## Animals and other research organisms

Policy information about studies involving animals; ARRIVE guidelines recommended for reporting animal research, and Sex and Gender in Research

| | |
|---|---|
| Laboratory animals | Male and female adult immunodeficient $Rag1^{-}/^{-}Il2rg^{-}/^{-}$ rats (RRG; 3–6 months old) were used for conventional and CCS-based organoid transplantations (founders from the Transgenesis Rat ImmunoPhenomic Platform, Nantes, France; in-house breeding). Adult immunodeficient NOD-SCID IL-2Rγnull (NSG) mice (3–6 months old) were used for conventional organoid experiments (purchased from the Comprehensive Mouse and Cancer Core Facility, Cincinnati, OH). Animals were housed in a barrier vivarium, with 14 hours light/10 hours dark cycle, approximately 37% humidity and temperature of 22.2°C, and handled in accordance with the NIH Guide for the Care and Use of Laboratory Animals. |
| Wild animals | No wild animals were used. |
| Reporting on sex | Findings did not apply to a specific sex; both sexes of rodents were included in the study. They served as bioreactors for organoid growth, animal tissues were not directly studied throughout the study. |
| Field-collected samples | No field-collected samples were used. |
| Ethics oversight | All animal work was completed with prior approval of the Institutional Animal Care and Use Committee at Cincinnati Children's Hospital Medical Center. |

Note that full information on the approval of the study protocol must also be provided in the manuscript.

## Plants

| | |
|---|---|
| Seed stocks | N/A |
| Novel plant genotypes | N/A |
| Authentication | N/A |
