# Supplementary material for: Large-scale and innervated functional human gut tissues for transplantation via transient spheroid confinement
Source: Nat Biomed Eng. 2026 May 22;10(10):2077–93. doi: 10.1038/s41551-026-01688-6 (PMC13672046; doi:10.1038/s41551-026-01688-6)
Supplement: Supplementary file 3 [file 41551_2026_1688_MOESM3_ESM.pdf]

nature portfolio

Peer Review File

Large-Scale and Innervated Functional Human Gut Tissues for Transplantation via Transient Spheroid Confinement

Corresponding Author: Dr Maxime Mahe

This manuscript has been previously reviewed at another journal. This document only contains information relating to versions considered at Nature Biomedical Engineering.

Version 0:

Decision Letter:

Dear Max,

Thank you for your revised manuscript, "Engineering Large-Scale and Innervated Functional Human Gut for Transplantation", and apologise for the time taken in getting back to you. As you know, we have consulted with two of the original reviewers from your previous submission, but only one of them - former Rev #3 - provided us with their comment (that you will find at the end of this message). However, and considering that the remaining points raised from former Revier #1 were minor, I am pleased to write that we shall be happy to publish the manuscript in *Nature Biomedical Engineering*.

We will be performing detailed checks on your manuscript, and in due course will send you a checklist detailing our editorial and formatting requirements. You will need to follow these instructions before you upload the final manuscript files.

Please do not hesitate to contact me if you have any questions.

Best wishes,

Valeria

__
Dr Valeria Caprettini
Senior Editor, *Nature Biomedical Engineering*

____

Reviewer #1 - former Reviewer #3 (Report for the authors (Required)):

The authors have addressed all points I raised. The paper is ready for publication.

Version 1:

Decision Letter:

Dear Max,

I am happy to inform you that your manuscript, "Large-Scale and Innervated Functional Human Gut Tissues for Transplantation via Transient Spheroid Confinement", has now been accepted for publication in *Nature Biomedical Engineering*.

Over the next few weeks, the figures will be checked for production quality, the text edited to ensure that it conforms to house style, and the manuscript typeset.

Our Articles are published about 40 days after the acceptance date (we recommend that you inform your institutional press office of this timeframe), and you will be notified of the actual publication date a few days in advance. Articles can be published any working day of the week, and are pushed live shortly after 10 am London time.

Publishing agreement. You will be asked to digitally sign a publishing agreement (grant of rights). After the signed publishing agreement has been received, the proofs of the article will be sent to you for review. If you have any queries during the production process, or you cannot meet the requested deadline for returning the proofs, please contact rjsproduction@springernature.com.

Nature Biomedical Engineering is a Transformative Journal. Authors may publish their research with us through the traditional subscription access route, or make their paper immediately open access through payment of an article-processing charge. More information about publication options is available.

You may need to take specific actions to comply with funder and institutional open-access mandates. If the work described in the accepted manuscript is supported by a funder that requires immediate open access (as outlined, for example, by Plan S) and your manuscript was originally submitted on or after January 1st 2021, then you should select the gold OA route. Authors selecting subscription publication will need to accept our standard licensing terms (including our self-archiving policies), and these will supersede any other terms that the author or any third party may assert apply to any version of the manuscript.

Acceptance of your manuscript is conditional on agreement, by all authors, with both our media embargo and confidentiality and pre-publicity policies. In particular, you may arrange your own publicity of the Article (for instance, through your institutional press office), as long as you ensure that journalists strictly adhere to the media embargo.

To assist you in disseminating the work, as soon as the Article is published you will be able to take advantage of the Springer Nature SharedIt initiative to generate a unique shareable link to the Article that will allow anyone (with or without a subscription) to read it. Recipients of the link who are subscribers will also be able to download and print the PDF.

Thank you for having submitted this work to *Nature Biomedical Engineering*.

Best wishes,

Valeria

__
Dr Valeria Caprettini
Senior Editor, *Nature Biomedical Engineering*

Open Access This Peer Review File is licensed under a Creative Commons Attribution 4.0 International License, which permits use, sharing, adaptation, distribution and reproduction in any medium or format, as long as you give appropriate credit to the original author(s) and the source, provide a link to the Creative Commons license, and indicate if changes were made.
In cases where reviewers are anonymous, credit should be given to 'Anonymous Referee' and the source.
The images or other third party material in this Peer Review File are included in the article's Creative Commons license, unless indicated otherwise in a credit line to the material. If material is not included in the article's Creative Commons license and your intended use is not permitted by statutory regulation or exceeds the permitted use, you will need to obtain permission directly from the copyright holder.
To view a copy of this license, visit https://creativecommons.org/licenses/by/4.0/

NBME-25-2366

Author Comments in Blue

Reviewer #1 - former Reviewer #3 (Report for the authors (Required)):

The authors have addressed all points I raised. The paper is ready for publication.

We thank the reviewer for their thoughtful comments throughout the entire revision process and are pleased that our revisions have addressed their concerns.
